# Landscape of Somatic and Age‐Related Pathogenic Structural Variations in Hepatocellular Carcinoma Revealed by Long‐Read Sequencing

**DOI:** 10.1002/mco2.70570

**Published:** 2026-01-13

**Authors:** Zhewen Wei, Yinghao Cao, Hongchao Liu, Mei Liu, Bolun Zhang, Jianming Ying, Jianqiang Cai, Xinyu Bi, Jianjun Zhao, Jianguo Zhou, Zhiyu Li, Zhen Huang, Jianmei Liu, Xueyan Lv, Zhiwen Luo, Zhicheng Wei, Xiaoshi Zhang, Yi Yang, Yiqiao Deng, Yanjiang Yin, Jinghua Chen, Junbo Liang, Xiaoyue Wang, Yefan Zhang, Hong Zhao

**Affiliations:** ^1^ Department of Hepatobiliary Surgery National Cancer Center/National Clinical Research Center for Cancer/Cancer Hospital Chinese Academy of Medical Sciences and Peking Union Medical College Beijing China; ^2^ Key Laboratory of Gene Editing Screening and R&D of Digestive System Tumor Drugs Chinese Academy of Medical Sciences and Peking Union Medical College Beijing China; ^3^ Center For Bioinformatics Institute of Basic Medical Sciences Chinese Academy of Medical Sciences School of Basic Medicine Peking Union Medical College Beijing China; ^4^ Department of Laboratory Medicine Peking University Third Hospital Beijing China; ^5^ Laboratory of Cell and Molecular Biology & State Key Laboratory of Molecular Oncology National Cancer Center/National Clinical Research Center for Cancer/Cancer Hospital Chinese Academy of Medical Sciences and Peking Union Medical College Beijing China; ^6^ Department of Hepatobiliary Surgery Aerospace Center Hospital Peking University Aerospace School of Clinical Medicine Beijing China; ^7^ Department of Pathology State Key Laboratory of Molecular Oncology National Cancer Center/National Clinical Research Center for Cancer/Cancer Hospital Chinese Academy of Medical Sciences and Peking Union Medical College Beijing China; ^8^ State Key Laboratory of Common Mechanism Research for Major Diseases Department of Biochemistry and Molecular Biology Institute of Basic Medical Sciences Chinese Academy of Medical Sciences School of Basic Medicine Peking Union Medical College Beijing China; ^9^ State Key Laboratory of Common Mechanism Research for Major Diseases Center For Bioinformatics National Infrastructures for Translational Medicine Institute of Clinical Medicine Peking Union Medical College Hospital Chinese Academy of Medical Science and Peking Union Medical College Beijing China

**Keywords:** hepatitis B virus integration, hepatocellular carcinoma, long‐read sequencing, somatic structural variations, younger ones

## Abstract

Hepatocellular carcinoma (HCC) was characterized by a highly complex genome, with structural variations (SVs) playing a significant role in its development. In this study, we employed Oxford Nanopore Technology long‐read sequencing in paired tumor and adjacent normal liver tissues from 74 Chinese HCC patients to thoroughly characterize the landscape of somatic SVs. Our analysis revealed that somatic SVs were more prevalent in hepatitis B virus (HBV)‐related HCC, with chromosome 1 emerging as a major hotspot, and several members of the chromosome 1 open reading frame (C1orf) family genes expression level exhibited significant age‐related difference. Notably, HBV‐related HCC cases exhibited a higher frequency of deletions, particularly among younger ones (≤ 35 years old). In addition, we observed an increased burden of HBV integration events in younger ones. Remarkably, the divergent‐paired related homeobox (*DPRX*) loci was identified as a novel gene for HBV integration in younger patients. Together, these findings delineated the somatic SV landscape in HCC and underscored age‐associated HBV‐related genomic alterations as key pathological features of hepatocarcinogenesis.

## Graphical abstract

This study leveraged long‐read sequencing (LRS) to provide a comprehensive analysis of structural variations (SVs) in the human hepatocellular carcinoma (HCC) genome. Somatic SVs were found to be more prevalent in HBV‐related HCC, with chromosome 1 emerging as the primary hotspot for these alterations. Notably, HBV‐related HCC exhibited a significantly higher frequency of deletions, particularly among younger patients (≤ 35 years old). *TERT* and *DPRX* loci were identified as recurrent hotspots for HBV integration. In addition, younger individuals showed a greater burden of HBV integration.

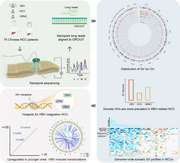

## 1 INTRODUCTION

Primary liver cancer ranked as the sixth most diagnosed cancer and the third leading cause of cancer‐related death worldwide [1]. Among its subtypes, hepatocellular carcinoma (HCC) was the most prevalent form. Well‐established risk factors for HCC included chronic infection with hepatitis B virus (HBV) or hepatitis C virus (HCV), exposure to aflatoxin B1, chronic alcohol consumption, and metabolic syndrome [2]. Globally, the incidence and mortality rates of liver cancer exhibit a male‐to‐female ratio of approximately 2.3:1, and this disparity is further pronounced in East Asia and China, where this ratio escalates to approximately 3:1 [1, 3]. Notably, HBV infection alone contributes to over 80% of HCC cases in China, where HBV integration reshapes genomic structures and promotes hepatocarcinogenesis early in life [4, 5].

These diverse etiologic factors were intertwined with genomic and molecular milieu, which played a crucial role in the initiation and progression of HCC [6]. Among the key genomic alterations, structural variation (SV) was one of the key pathological genetic components, which were commonly defined as genetic variants over 50 base pairs in size, including deletions (DELs), insertions (INSs), duplications/amplifications (DUPs), inversions (INVs), translocations, chromosomal losses and gains, and more complex DNA rearrangement patterns [7, 8]. Unlike point mutations, it is worth noting that somatic SVs can amplify oncogenes, disrupt tumor suppressor genes, fuse with cancer‐related genes, or repurpose noncoding genes to dysregulate gene expression, facilitating cancer genome evolution [9]. HBV further complicates this landscape by integrating into the host genome, inducing chromosomal instability that drives hepatocarcinogenesis [10]. The genomic landscape of HCC was remarkably complex, with somatic SVs playing an important role in its development and progression.

Previous studies utilizing short‐read sequencing (SRS) have substantially expanded our understanding of genomic alterations in HCC, particularly in single‐nucleotide variants (SNVs) and short insertions or deletions (Indels) [11, 12, 13, 14, 15]. However, spectrum and pathogenesis of SVs and their relationships with exogenous HBV integration in HCC remained largely underexplored due to the limited SRS technology, which made resolving long segments and complex genomic rearrangements difficult. Recent advances in long‐read sequencing (LRS), such as Oxford Nanopore Technology, now enable precise detection of SVs genome‐wide [16]. LRS has recently emerged as a powerful tool that enables more accurate detection of large SVs, repetitive sequences, and full‐length HBV integration events in human HCC genome [9, 17, 18]. We successfully captured full‐length HBV integration sequences (median length ∼10 kb) using LRS method and found that HBV‐mediated genomic rearrangements were significantly correlated with young patients (≤ 35 years old) [5]. Patients aged ≤ 35 years were defined as younger patients, consistent with previous studies that used this cutoff to characterize other solid tumors [19, 20]. Compared with short‐read approaches, LRS greatly enhanced SV detection, as they considerably facilitating de novo genome assembly and mapping repetitive or other problematic regions [21].

In this study, we employed LRS to delineate the comprehensive landscape of somatic SVs in HCC, focusing on age‐associated genomic alterations driven by HBV integration. We identified chromosome 1 as a major SV hotspot, with deletions disproportionately prevalent in HBV‐related HCC, particularly among younger patients. Notably, we uncovered divergent‐paired related homeobox (*DPRX*) as a novel HBV integration site enriched in younger individuals and revealed age‐dependent transcriptional dysregulation linked to TGF‐β signaling pathway and chromosome 1 open reading frame (C1orf) family genes. Our findings provide valuable insights into the genomic architecture of HCC, uncover novel features of HBV integration. Furthermore, these results reveal age‐specific molecular vulnerabilities, highlighting potential biomarkers and therapeutic targets for early‐onset HBV‐related HCC.

## 2 RESULTS

### 2.1 Characterization of Samples and Identification of Somatic SVs

**FIGURE 1:**
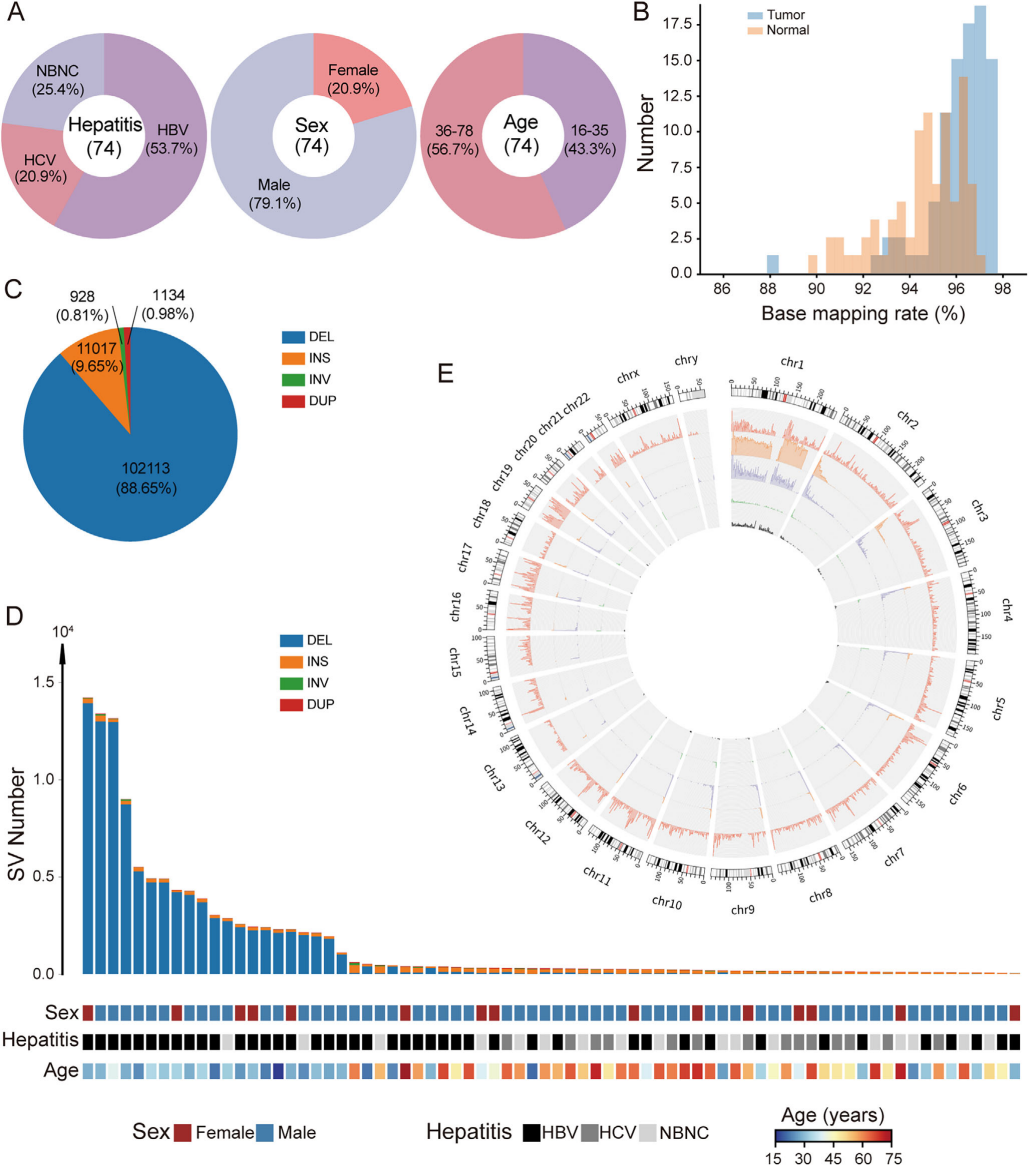
Systematic analysis of somatic SVs in HCC. (A) Overview of clinical and biological characteristics of the patient cohort. (B) Distribution of base‐mapping rates of clean read aligned to the human reference genome GRCh37. (C) Proportions of different structural variation (SV) types identified across all hepatocellular carcinoma (HCC) samples. (D) Number of somatic SVs per sample, stratified by sex, hepatitis status, and age group. (E) Circos plots illustrating chromosomal distribution of SVs and gene density: outermost ring shows GRCh37 reference gene locations; inner rings represent the distribution of deletions (DELs), insertions (INSs), duplications (DUPs), and inversions (INVs), respectively.

Tumor and matched adjacent normal liver tissue samples were collected from 74 Chinese HCC patients for sequencing. Among these individuals, the results of serological tests demonstrated that 43 (53.7%) had HBV infection, 14 (20.9%) had HCV infection, and 17 (25.4%) were negative for both HBV and HCV (NBNC) infection. Additionally, 59 (79.1%) were males and 15 (20.9%) were females, whose ages ranged from 16 to 78 years old, with a median age of 45 years old; 32 (43.3%) were younger than or equal to 35 years, and 42 (56.7%) were older than 35 years (Figure 1A and Table S1).

We performed whole‐genome LRS using Oxford Nanopore Technology on all tissues, generating an average of 48.8 Gb of sequence data per sample, with an average read length of 9,516 bp (Table S2). Then, we aligned all clean reads to human reference genome GRCh37 using the long‐read mapper NGLMR [22]. The median mapping rate was 96.0% for tumor tissues (range, 87.9%–97.4%) and 94.6% for normal tissues (range, 89.6%–96.9%, Figure 1B). The genetic variations of DELs, INSs, INVs, and DUPs were identified using the SV caller Sniffles, with a minimum indel length threshold of 50 bp and a minimum of two supporting reads [22]. Due to the lack of standardized criteria for somatic SVs identification in current LRS‐based studies, we developed a custom pipeline to define somatic SVs, which met both of the following conditions: (1) The SV must be present in the tumor sample but absent in the matched normal tissue from the same individual; (2) The corresponding SV region and its flanking sequences in the normal sample must contain at least two supporting reads, confirming the absence of the SV in normal tissue.

### 2.2 Systematic Analysis of Somatic SVs in HCC

In total, 115,192 high‐confidence somatic SVs were identified across the 74 HCC patients, including 102,113 (88.65%) DELs, 11,017 (9.65%) INSs, 928 (0.81%) INVs, and 1,134 (0.98%) DUPs (Figure 1C). The number of somatic SVs per sample varied widely, ranging from 44 to 14,228 with a median of 292, and the distribution of SV types also differed across individuals. Notably, the average number of deletions in HBV‐related HCC was approximately fivefold higher than in NBNC‐related HCC and 50‐fold higher than in HCV‐related HCC (Figure 1D). In contrast, insertions were more prominent in HCV‐related HCC, with their average number being three times higher than that of deletions (Table S3). Interestingly, both deletions and insertions were most frequently observed on chromosome 1, suggesting this chromosome as a potential hotspot for SVs in HCC (Figure 1E and Table S4).

### 2.3 Genome—Wide Somatic SV Profiles Associated With HCC

**FIGURE 2:**
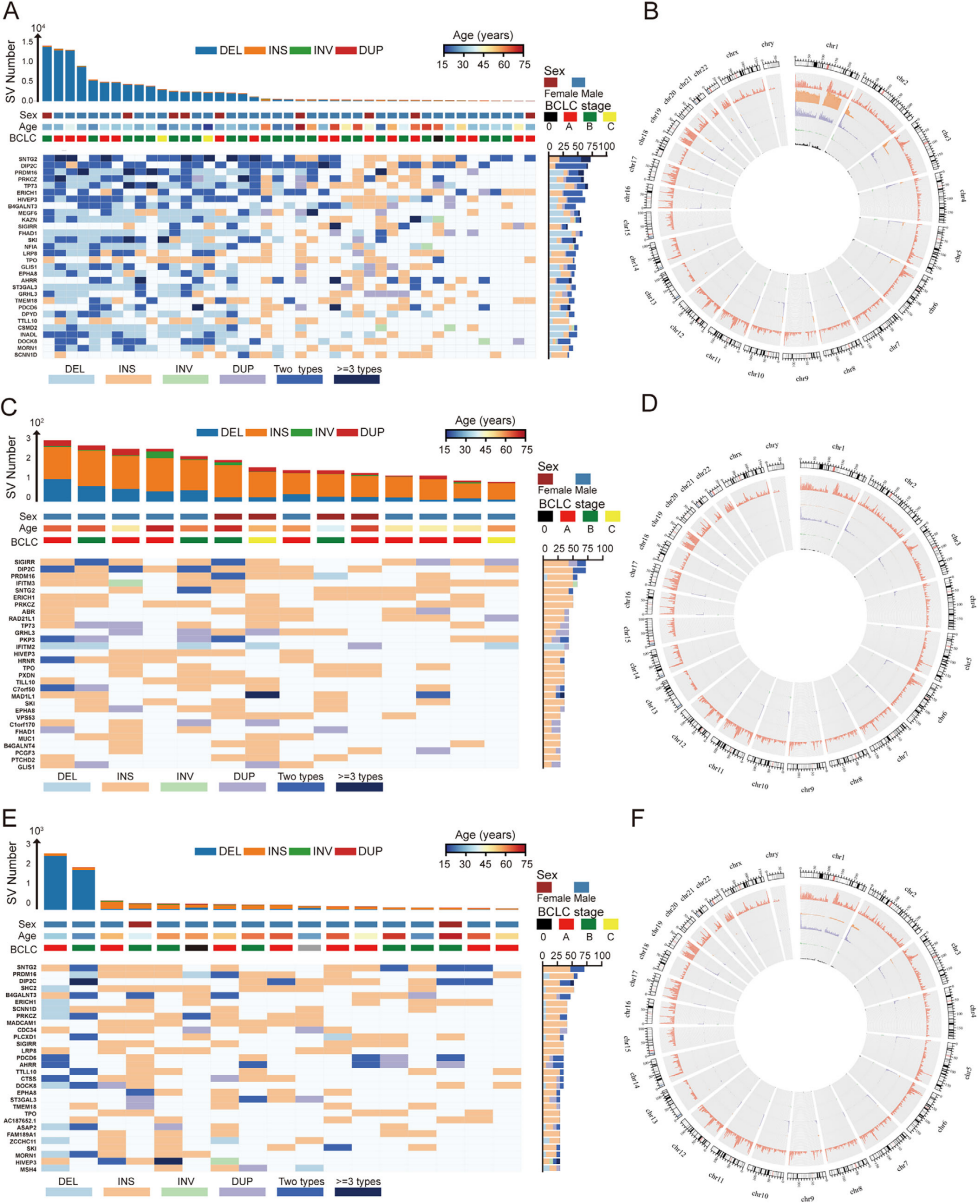
Genome‐wide somatic SV profiles associated with HCC. Heatmaps showing gene‐level somatic SV profiles in (A) HBV‐related HCC, (C) HCV‐related HCC, and (E) NBNC‐related HCC, respectively. SV types are color coded. Circos plots displaying the number of genes affected by SVs and their chromosomal distribution in (B) HBV‐related HCC, (D) HCV‐related HCC, and (F) NBNC‐related HCC, respectively (outermost ring shows GRCh37 reference gene locations; inner rings represent the distribution of DELs, INSs, DUPs, and INVs, respectively).

To systematically evaluate the potential functional impact of somatic SVs, we conducted a genomic overlap analysis using BEDTools to identify SVs intersecting with RefSeq gene annotations (GRCh37). Specifically, we quantified exonic disruptions and gene‐level overlaps (Figure S1A). We next described the functional outcomes of HBV‐related SVs across different genomic regions. SVs were categorized by variant type, and their genomic attributes are shown in Figure 2A, and chromosomal distribution of each somatic SVs is visualized using a circos plot (Figure 2B). Chromosome 1 appeared to be the top hotspot for SVs that were attributed to DELs and INSs. Genomic loci frequently impacted most by SVs, characterized by the presence of more than two distinct SV breakpoint types, included *SNTG2*, *DIP2C*, *PRDM16*, *TP73*, *ERICH1*, *HIVEP3*, *B4GALNT3*, *MEGF6*, *KAZN*, and *SIGIRR*. A distinctive functional SVs landscape was observed in HCV‐related HCC, predominantly involving INSs. Frequently affected genes in this group included *SIGIRR*, *DIP2C*, *PRDM16*, *IFITM3*, *SNTG2*, *ERICH1*, *PRKCZ*, *ABR*, *RAD21L1*, and *TP73* (Figure 2C,D). In NBNC‐related HCC, SVs were primarily insertions, except for two patients who exhibited high numbers of deletions. The SV‐affected gene cohort in this subgroup included *SNTG2*, *PRDM16*, *DIP2C*, *SHC2*, *B4GALNT3*, *ERICH1*, *SCNN1D*, *PRKCZ*, *MADCAM1*, and *CDC34* (Figure 2E,F).

### 2.4 Different Types of Somatic SVs and Related Repeats

**FIGURE 3:**
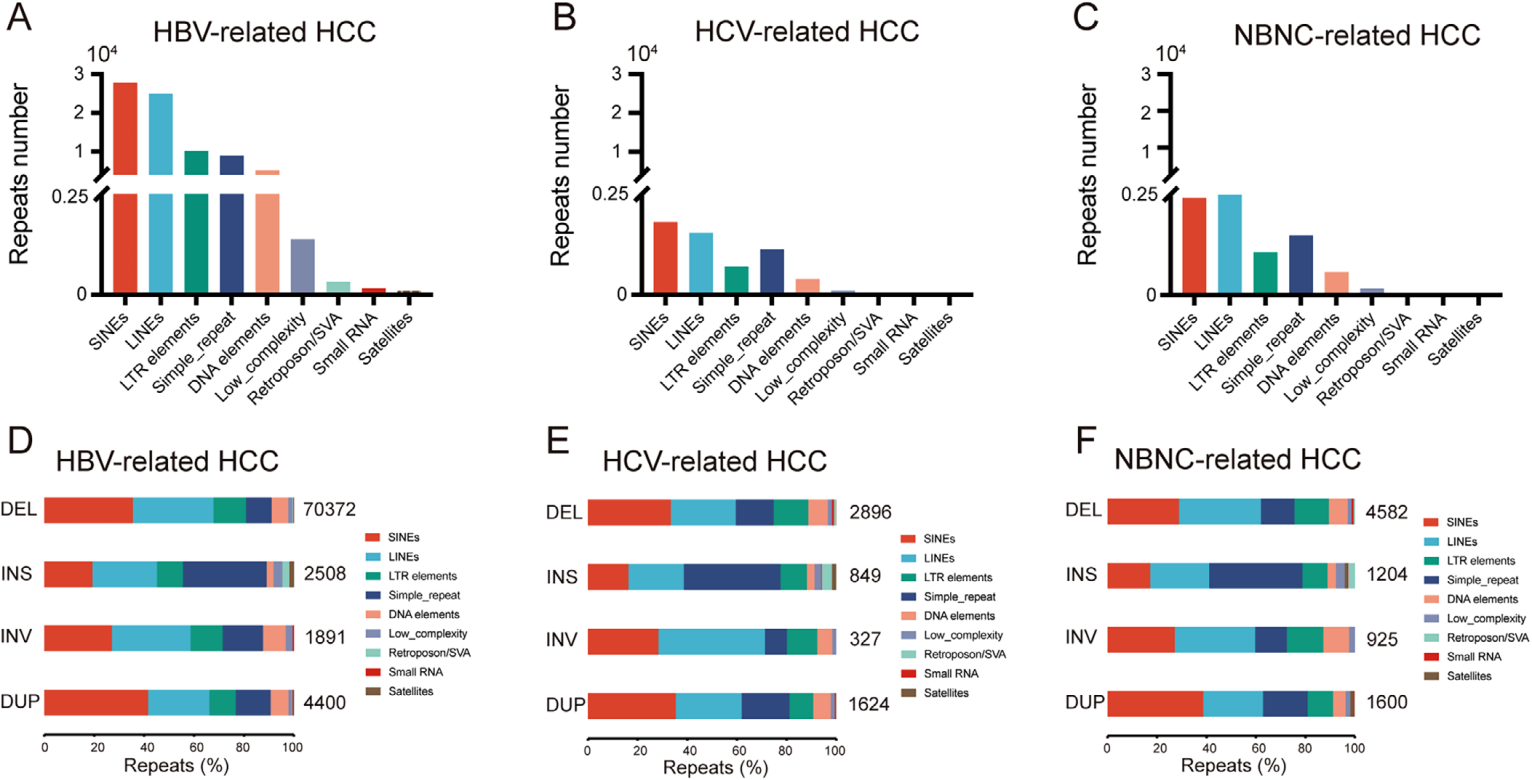
Repeat element composition associated with different types of somatic SVs in HCC subtypes. Repeat element composition in (A) HBV‐related HCC, (B) HCV‐related HCC, and (C) NBNC‐related HCC samples. Proportions of various repeat elements in each SV type in (D) HBV‐related HCC, (E) HCV‐related HCC, and (F) NBNC‐related HCC samples.

Previous studies have suggested that deletions and insertions associated with repeated DNA sequences may reflect distinct mechanisms of somatic SVs formation [23, 24]. We annotated all deletions and insertions using RepeatMasker, which served as a reference for identifying repeat‐associated elements [25]. We first quantified the total number of repetitive elements in each cohort. In the HBV cohort, the total number of repetitive elements was approximately 74,888 (Figure 3A). The HCV (Figure 3B) and NBNC (Figure 3C) cohort revealed that the total count of repetitive elements was approximately 3620 and 6795. Notably, short interspersed nuclear elements (SINEs) and long interspersed nuclear elements (LINEs) were the most predominant repeats in each cohort. SVs containing repetitive DNA sequences were significantly more prevalent in HBV‐infected patients compared to those with HCV‐ or NBNC‐related HCC. We next analyzed the proportional contribution of various repetitive elements to different types of SVs. Specifically, DEL, INV, and DUP events were frequently associated with SINEs and LINEs, whereas INS events were predominantly linked to simple repeats and LINEs in the three cohorts (Figure 3D–F, Table S5).

### 2.5 HBV Integration in HBV‐HCC

**FIGURE 4:**
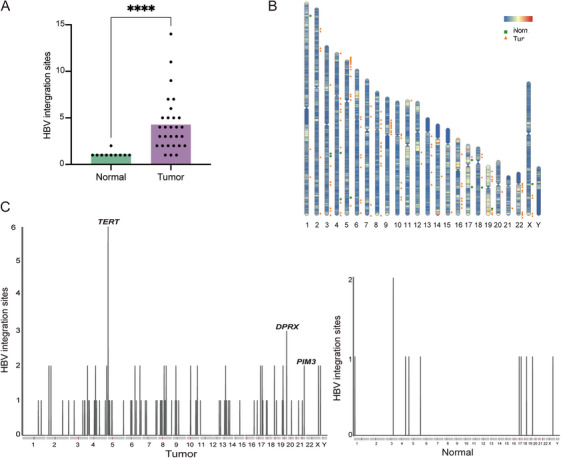
HBV integration profiles in HBV‐related HCC. (A) Comparison of number of HBV integration sites in tumor and normal tissues. Statistical significance was evaluated using the Wilcoxon rank‐sum test. (B) Chromosomal distributions of HBV integration sites in tumor and normal tissues. (C) Recurrent affected genes by HBV integrations in tumor and normal tissues.

HBV integration has been appreciated as a catalyst for chromosomal rearrangements in HCC that display differential patterns associated with designated functional impacts between tumor and normal tissues [26, 27]. Here, 124 HBV integration sites were identified in tumor tissues, a number significantly higher than the 11 HBV integration sites observed in normal tissues (*p* < 0.0001, Wilcoxon rank‐sum test, Figure 4A). The chromosomal distribution of these HBV integration sites is depicted in Figure 4B. Among these, HBV integration events near telomerase reverse transcriptase (*TERT*) gene, a well‐known target of HBV integration, were observed six times in tumor tissues across five patients. Additionally, the divergent‐paired related homeobox (*DPRX*) gene exhibited three clonal integration events in three patients, while the serine/threonine‐protein kinase pim‐3 (*PIM3*) gene exhibited two events in a single patient (Figure 4C).

### 2.6 Age‐Associated Somatic SVs in HBV‐HCC

**FIGURE 5:**
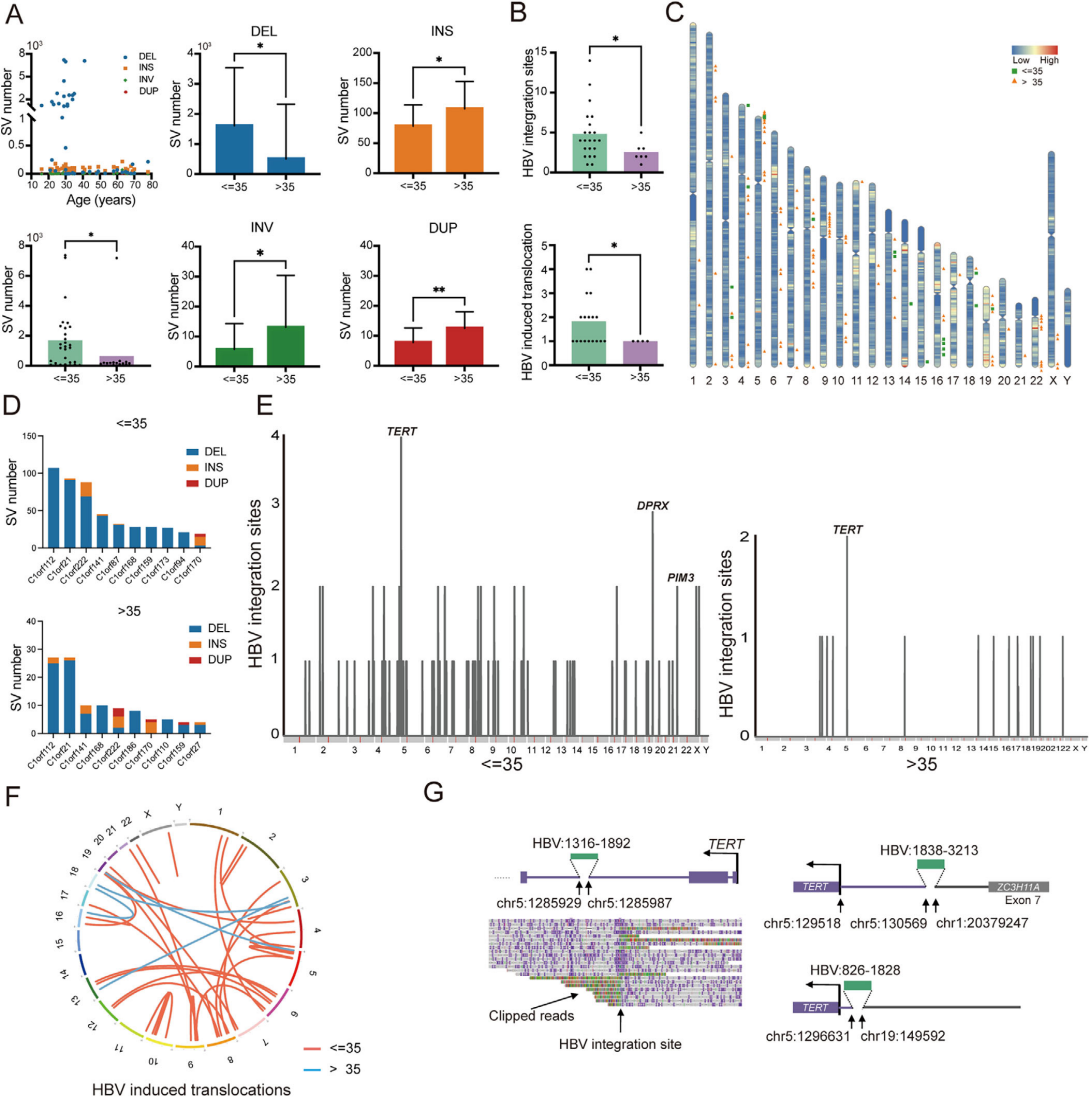
Age‐associated somatic SVs and HBV integration patterns in HBV‐related HCC. (A) Distribution of somatic SV types including DELs, INSs, INVs, and DUPs in patients aged ≤ 35 and > 35 years. (B) Comparison of the number of HBV integration sites and HBV‐induced translocations between the two age groups. (C) Chromosomal distributions of HBV integration sites in the ≤ 35 and > 35 groups. (D) SV number in chromosome 1 open reading frame (C1orf) family genes in the ≤ 35 and > 35 groups. (E) Chromosomal distribution of recurrent HBV integration sites in individual patients. (F) Recurrent HBV‐induced translocations mapped across human chromosomes. (G) Representative examples of HBV‐induced translocations near the TERT locus. Statistical significance was assessed using unpaired *t*‐test (A and D) and the Wilcoxon rank‐sum test (B).

In HBV‐related HCC cohort, we observed that somatic SVs, particularly deletions, displayed a marked age bias, occurring more frequently in younger patients (28 patients ≤ 35 years old, *p* < 0.05, unpaired *t*‐test, Figure 5A), suggesting an age‐dependent pathogenic role of SVs in HBV‐driven hepatocarcinogenesis. Importantly, younger individuals also exhibited a significantly higher number of HBV integration sites and induced translocations (*p* < 0.05, Wilcoxon rank‐sum test, Figure 5B). Chromosomal distributions of HBV integration sites are shown in Figure 5C. Notably, genes from the chromosome 1 open reading frame (C1orf) family harbored a greater number of SVs in younger patients (*p* < 0.0001, unpaired *t*‐test, Figure 5D). We next focused on the genes that were either directly disrupted by HBV integration or located near integration breakpoints. In younger patients, recurrent HBV integrations were detected in multiple samples, including *TERT* (n = 3 samples, 4 events), *DPRX* (n = 3 samples, 3 events), and *PIM3* (n = 1 sample, 2 events). In contrast, the counterparts (15 patients >35 years old) exhibited recurrent events in *TERT* (*n* = 2 samples, 2 events) and zero events in *DPRX* (Figure 5E). The frequency of HBV integration near the *TERT* gene did not differ significantly between the two groups (10.7% vs. 13.3%, *p* > 0.99, Fisher's exact test). HBV integration with chimeric HBV‐human junction sequences near *DPRX* was observed in three patients, and two HBV integration sites without chimeric junctions were identified near *PIM3* from a single patient (Figure S1B). HBV integration sites near *DPRX* and *PIM3* were validated by Sanger sequencing (Figure S1C,D). To gain further insights into the relationship between HBV integration and SV formation, we performed detailed analysis based on multiple alignments of the LRS data. A higher number of HBV‐induced translocations were identified in the younger group (Figure 5F). Strikingly, *TERT* was preferentially affected by these genomic alterations (Figure 5G).

### 2.7 Age‐Associated HBV‐HCC Transcriptional Landscape

**FIGURE 6:**
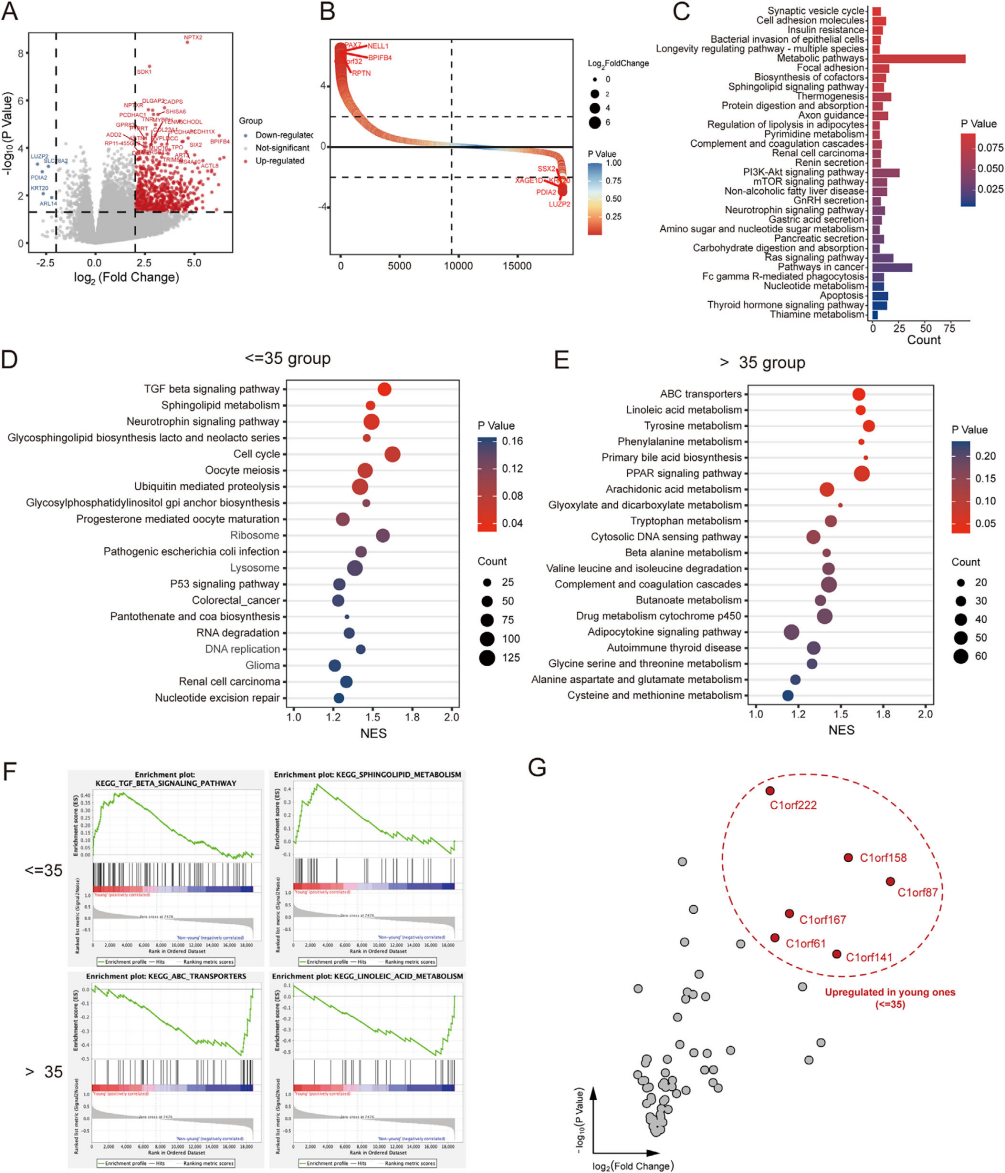
Age‐associated transcriptional landscape of HBV‐related HCC. (A) Differentially expressed genes (DEGs) between patients age ≤ 35 and > 35 years. (B) Rank‐order plot of DEGs in the two age groups. (C) KEGG pathway enrichment analysis of age‐associated DEGs. (D and E) Gene set enrichment analysis (GSEA) of the upregulated genes in (D) age ≤ 35 years group and (E) age > 35 years group. (F) Representative hallmark gene sets enriched in each group, as identified by GSEA. (G) Differential expression of C1orf family genes between the ≤ 35 and > 35 age groups.

Lastly, we conducted an analysis of the transcriptional profiles of HBV‐related HCC in relation to patient age. A total of 1188 age‐associated differentially expressed genes (DEGs) were identified, including 1128 upregulated and 60 downregulated genes in the younger cohort compared to the counterpart group (Figure 6A,B). To explore the functional relevance of these DEGs, we performed Kyoto Encyclopedia of Genes and Genomes (KEGG) pathways enrichment analysis using Gene Set Enrichment Analysis (GSEA). The SV‐associated gene module included key regulators of cancer‐related pathways, such as apoptosis, and the Ras, mTOR, and PI3K‐Akt signaling pathways (Figure 6C). Notably, the TGF‐β signaling pathway, sphingolipid metabolism, and related processes were significantly enriched in the younger group (Figure 6D, F). In contrast, the group aged over 35 years showed enrichment of dysregulated metabolic pathways, particularly those involved in linoleic acid regulation and activity of ABC transporters (Figure 6E,F). Moreover, several members of the C1orf gene family—including *C1orf61*, *C1orf222*, *C1orf158*, *C1orf87*, *C1orf167*, and *C1orf141*—exhibited significantly differential expression between the two age groups, further supporting the age‐dependent transcriptional divergence in HBV‐related HCC (Figure 6G).

## 3 DISCUSSION

Compared with SRS technologies, long‐read platforms are currently considered the most effective approach for comprehensive detection of somatic SVs, particularly large and complex events [28, 29]. In the present study, we established a stringent identification strategy and developed a customized analytical pipeline to accurately define somatic SVs. By applying rigorous filtering criteria, we effectively reduced the false‐positive rate and generated a high‐confidence set of somatic SVs. This approach enabled a comprehensive analysis of somatic SVs in HCC patients with diverse hepatitis infection backgrounds, including HBV, HCV, and NBNC‐related subtypes.

Compared with previously published LRS datasets from Icelandic [17] and Chinese [18] populations, our study identified a substantially higher number of deletions, both in absolute count and as a proportion of total SVs. Notably, an exceptionally high somatic SV burden, particularly of deletion, was observed in HBV‐related HCC, significantly exceeding that in HCV‐ or NBNC‐related subtypes. The average number of deletions in HBV‐related HCC patients was markedly greater than in patients with either HCV‐ or NBNC‐related HCC. These findings highlight deletions as the predominant SV type in HBV‐related HCC, consistent with previous observations from a Japanese cohort [30], and to a lesser extent, in NBNC‐related cases. Epidemiological studies have reported that individuals infected with HBV have a 10‐ to 30‐fold increased risk of developing HCC compared to uninfected individuals [31]. Based on our results, we proposed that the high burden of deletions may represent a key genomic mechanism contributing to this elevated cancer risk in HBV‐infected patients. Moreover, copy number gain of chromosome 1q, a region recurrently affected by deletions in our dataset, is one of the most frequent genomic alterations reported in HCC based on SRS data, further supporting its role in hepatocarcinogenesis [32].

The present study focused specifically on large SVs (> 50 bp), whose functional consequences often differ fundamentally from those of smaller variants. Unlike SNVs or small indels, large SVs can disrupt entire exons, alter gene structure, or lead to copy number changes, resulting in more pronounced but less predictable effect. Due to these characteristics, conventional variant effect predictors such as ANNOVAR or VEP, which are optimized for small variants, are less suitable for SV interpretation. Therefore, we employed BEDTools to evaluate genomic overlaps, particularly between SVs and gene annotations. SVs intersecting with exonic regions are likely to cause significant gene dysfunction, potentially affecting coding sequences as well as regulatory regions. This supported the notion that SV burden was correlated with functional impact. While the sets of genes mostly frequently affected by SVs differed across HBV‐, HCV‐, and NBNC‐related HCC, several genes including *SNTG2*, *DIP2C*, *PRDM16*, and *ERICH1* were recurrently affected in all three subtypes. Among the identified genes, *PRDM16* is relatively well studied and has been linked to HCC progression through upregulation and aberrant activation of the Notch signaling pathway [33]. *DIP2C* is considered a potential tumor promotor, possibly involved in epigenetic regulation and DNA methylation [34]. In contrast, *SNTG2*, *DIP2C*, and *ERICH1* remain poorly characterized in HCC. However, their recurrent disruption by SVs in our dataset suggests a potential functional role in hepatocarcinogenesis and warrants further investigation. Together, these findings not only suggested a significant contribution of somatic SVs to HCC carcinogenesis but also highlighted distinct SV patterns and functional impacts associated with different hepatitis viral infections.

The presence of repetitive sequences in the genome was closely linked to the formation of SVs. These elements not only served as a common genomic background for SVs but also actively influenced their occurrence and type. Repetitive elements, particularly SINEs and LINEs, were known to contribute to genomic instability and were frequently implicated in the formation of deletions and insertions [30]. Therefore, we performed repeat annotation of SV sequences using RepeatMasker. The results revealed a strong correlation between the number of repeats and the number of SVs, with SINEs and LINEs contributing substantially to both deletions and insertions. Notably, although multiple types of repetitive elements were found in both SV types, their distribution patterns differed, suggesting distinct mechanisms of SV formation. These findings highlighted the pathogenic relevance of deletions and insertions, particularly in the context of hepatitis virus‐associated HCC. In HBV‐related HCC, we observed that SV‐affected genes showed increased expression in regions of deletions and insertions on chromosome 1, suggesting functional consequences linked to SV burden. Interestingly, chromosome 1 exhibited the highest frequency of duplications, while chromosome 4 had the greatest number of deletions—a pattern that differed significantly from previous reports [35]. This observation underscored potential HBV‐specific chromosomal vulnerabilities, warranting further mechanistic investigation.

C1orf family genes exhibited a high frequency of SV in HBV‐related HCC, particularly among younger patients, it probably related to HBV integration. Moreover, the expression levels of several C1orf genes significantly differed between the two diverse age groups, and C1orf61, C1orf87, and C1orf141 showed age‐dependent differences in both SV burden and transcript expression levels. These findings suggested that SV‐mediated alterations in these genes may contribute to transcriptional dysregulation in an age‐specific HBV integration manner. Studies have reported that C1orf61 promoted HCC metastasis by inducing cellular epithelial‐mesenchymal transition (EMT) via the STAT3 and Akt cascade pathways, and elevated expression of C1orf61 has been associated with enhanced therapeutic efficacy of the anticancer agent sorafenib in patients with HCC [36]. The remaining C1orf family gene‐related mechanisms deserved further investigation in the future. While the specific types of SVs responsible for expression changes remained unclear, it is plausible that different SV mechanisms such as deletions, insertions, or duplications, had distinct regulatory consequences. For instance, gene deletions may lead to compensatory upregulation of homologous or functionally related genes via mechanisms such as negative feedback regulation, transcriptional activation of regulatory elements, or epigenetic modulation [37, 38]. These results further confirmed the differential impact of SVs on HBV‐related HCC in an age‐dependent manner, suggesting the possibility of the development of more therapeutic‐specific regimens by targeting age‐associated differential functions.

Previous studies reported that HBV infection was associated with chromosomal instability in both cancerous and noncancerous liver tissues and that HBV integration acted as a key driver of chromosomal rearrangements in HCC [10]. In our study, we also observed a significantly higher number of HBV integration sites in tumor tissues compared to matched normal tissues, indicating a tumor‐specific bias that may contribute to HCC carcinogenesis. Younger patients appeared to have increased burdens of HBV integration and HBV‐induced translocations, suggesting a potential age‐dependent vulnerability to viral genome incorporation and subsequent genomic instability. Importantly, the extent of HBV integration has been reported as an independent prognostic factor in HBV‐related HCC, particularly in younger individuals, and was associated with poorer clinical outcomes [10, 39]. We speculated that early‐life HBV infection may lead to persistent integration of viral DNA into the host genome, which subsequently triggers structural genomic alterations, including somatic SVs, even in individuals receiving antiviral therapy. This hypothesis was further supported by clinical observations showing that HCC can develop after serological clearance of HBsAg, with residual HBV DNA integrated into the host genome [40]. Such persistent integrations likely contributed to the malignant transformation of hepatocytes, underscoring the pathogenic significance of HBV‐host genome interactions in the early stages of hepatocarcinogenesis.

The *TERT* and *DPRX* loci were the hotspots for HBV integration in our study. *TERT* was the first reported recurrent gene affected by HBV integration, as well as it was also the most common one in HCC samples, ranging from nearly 20% to 36% [41]. The frequence of *TERT* (5/43, 11.6%) affected by HBV integration in our study was lower than previous reports. *TERT* was the most frequently affected locus in two age groups, but the frequency of occurrence did not differ significantly between the two groups. The *DPRX* loci (3/28, 10.7%) emerged as hotspot for HBV integration in younger patients, it represented novel HBV integration site identified in this study. It is a poorly characterized homeobox gene, with limited functional annotation in the current literature. However, homeobox genes were broadly implicated in developmental regulation. *DPRX* encoded a homeobox DNA‐binding protein implicated in early embryonic development [42]. The identification of HBV integration near DPRX may suggest a potentially novel role for this gene in liver cancer biology, warranting further investigation into its regulatory function and contribution to oncogenesis. *PIM3* gene represented the multiple HBV integration sites in a single patient. PIM3 was a member of the PIM family of oncogenic serine/threonine kinases, and it has established roles in cell proliferation, immune regulation, and tumor development [43, 44]. It has been shown to promote tumor progression in various cancers, including HCC, primarily through the activation of oncogenic signaling pathways such as JAK/STAT and MAPK [45]. The mTOR and PI3K‐Akt signaling pathways were closely related to cell proliferation and tumor angiogenesis [46]. In our study, TGF‐β signaling and cell cycle regulation pathways were preferentially enriched in the younger HCC cohort, both of which were implicated in tumor invasion, metastasis, and apoptosis, as well as closely interconnected with PI3K‐Akt signaling [47]. Mutations in TGF‐β pathway components have also been reported to show age‐related associations in colorectal cancer [48]. The enrichment of these pathways in younger patients may suggest a unique molecular program that may underlie early‐onset disease biology. These tumors may exhibit unique biological behaviors compared to those in older patients. For example, targeting the TGF‐β pathway was currently under investigation in multiple cancer types [49], and sphingolipid metabolism dysregulation was also observed in younger individuals, which offered additional therapeutic potential. Taken together, these findings indicated that such pathways may serve as age‐specific biomarkers or therapeutic targets in HBV‐related HCC. Furthermore, a comprehensive understanding of SV‐driven genes enabled by LRS will be essential for elucidating key oncogenic mechanisms and identifying novel therapeutic opportunities in HCC.

There were several limitations in our study. As we applied a cut‐off of >50 bp to minimize false positives given the relatively high error rates of LRS, SVs smaller than 50 bp were excluded from this study, which may miss some SVs. In addition, the sample size, particularly for the HCV‐ and NBNC‐related HCC groups, and the depth of genomic coverage were suboptimal, which may limit the statistical power of some analyses. Due to the small cohort sizes, multiple testing correction was not applied, and the current findings should be interpreted as exploratory. Future studies with larger, well‐balanced cohorts and deeper sequencing coverage will be essential for the robust identification of individual germline and subclonal SVs. Moreover, although not addressed in this study, the epigenetic consequences of SVs, including alterations in DNA methylation and noncoding regulatory elements, represented an important avenue for future research to fully elucidate the pathogenetic impact of structural rearrangements in HCC.

## 4 CONCLUSIONS

Our analysis revealed a high prevalence of complex somatic SVs in the HCC genome in the HCC genome, with deletions predominating in HBV‐infected patients, particularly in the younger cohort. Both somatic SV landscapes and HBV integrations in younger individuals differed markedly from those in non‐young patients. These findings provided novel insights into the age‐dependent molecular mechanisms of HBV‐driven hepatocarcinogenesis and may uncover potential therapeutic targets tailored to the unique biology of early‐onset HBV‐related HCC.

## 5 MATERIALS AND METHODS

### 5.1 Clinical Samples

We retrospectively retrieved clinical samples from a biobank that met the following criteria: Patients with primary HCC who had been treated with surgical resection were identified; HBV‐related patients who were HBsAg positive; HCV‐related patients who were anti‐HCV positive; and NBNC‐related patients who were HBsAg‐ and anti‐HCV‐negative. Inclusion criteria were as follows: (I) patients who underwent hepatectomy; (II) pathological diagnosis was HCC; and (III) tissue quality control was qualified. Exclusion criteria were as follows: (I) a history of prior cancer surgery; (II) combined with other therapy before or during surgery; (III) had missing data that made it impossible to be evaluated. The sample size was distributed among the three groups of HCC patients as much as possible. A total of 74 individuals were included in this study (59 males and 15 females), including 43 HBV‐related patients, 14 HCV‐related patients, and 17 NBNC‐related patients. All tumor and paired nontumor tissues were obtained after surgery and stored at −80°C. In each case, the diagnosis was supported by the results of histopathology examination conducted by a qualified pathologist.

### 5.2 DNA Extraction, Library Preparation, and Nanopore Sequencing

High‐molecular‐weight genomic DNA was prepared via the CTAB method, followed by purification with a QIAGEN Genomic Kit (Cat# 13343, QIAGEN) following the manufacturer's instructions. Ultralong DNAs were extracted via the SDS method without a purification step to prevent fragmentation of the DNA. The levels of degradation and contamination were monitored by visualizing DNA separated on 1% agarose gels. DNA purity was determined via a NanoDrop One UV‒Vis spectrophotometer (Thermo Fisher Scientific, USA). For all sequencing samples, the OD260/280 values were between 1.8 and 2.0, and the OD260/230 values were between 2.0 and 2.2. The DNA concentration was measured via a Qubit 4.0 fluorometer (Invitrogen, USA).

A total of 3–4 µg of DNA per sample was used for Oxford Nanopore Technology (ONT) library preparation. After the sample was qualified, a PippinHT system (Sage Science, USA) was used to select long DNA fragments. The ends of the DNA fragments were repaired in conjunction with A‐ligation via the NEBNext Ultra II End Repair/dA‐tailing Kit (Cat# E7546). The adapters in SQK‐LSK109 (Oxford Nanopore Technologies, UK) were introduced into further ligation, and the constructed library was quantified with a Qubit 4.0 fluorometer (Invitrogen, USA). For each sample, approximately 700 ng of library DNA was sequenced via a Nanopore PromethION sequencer (Oxford Nanopore Technologies, UK) by the Center of Grandomics (Wuhan, China).

### 5.3 Long‐Read Alignment and SV Assignment

The bam files were generated by aligning the Nanopore long reads to the GRCh37 human reference genome via NGMLR (version: 0.2.7) [22]. Sniffles (version:1.0.12) [22] were used to define the SV with the main parameters as follows: –report_BND –ignore_sd –minmapping_qual 20 –num_reads_report ‐1 –min_length 50 –min_support 1 –min_het_af 0.1 –genotype.

### 5.4 Somatic SV Verification and Analysis

In both tumor and nontumor tissues, identification was excluded when supported by fewer than two reads within the region; verified SVs were labeled with a “PASS” tag. In germline tissues, SVs longer than 1 Mb were discarded to mitigate false positive calls attributable to background noise. In tumor tissues, SVs are identified as precise SVs supported by at least two variant reads coupled with allele frequencies greater than 0.1.

For large‐scale identification of somatic SVs, we considered only uncomplicated SVs, such as deletions, insertions, duplications, and inversions within chromosomes. Somatic SVs were analyzed in two steps as follows. First, filtration was performed on the basis of the SV information of the germline tissues. For deletions, duplications, and inversions in tumors, instead of considering the overlap of those segments in germline tissues, we discarded segments in tumors directly when the same SV was found within 1000 bp adjacent to germline tissues. For insertions in tumors, we discarded segments when any insertion breakpoints within 300 bp could be found in germline tissues. Second, to minimize false positive identification of retained somatic SVs, we checked whether the same region of somatic SV in the paired germline tissue had reference sequence read support. In this process, SAMtools (version: 1.13) was used to extract the reference sequence reads of germline tissues near the region of the somatic SV, with the intersect function in BEDTools (version: 2.30.0) used to check for 50% overlap via the parameter ‐f 0.5. Somatic SVs were discarded if no reference sequence reads were found in the same region in paired germline tissue.

Furthermore, to reduce false positive investigations of the impact of SV on genes, we added a new filter for somatic SVs via a “normal panel” by merging the germline SVs of all the samples. The panel was created by merging all the identified germline SVs if they were within 1000 bp (deletion, duplication, and inversion) or 300 bp (insertion). Somatic SVs were filtered if any overlap region could be found compared with the normal panel. After this filtration, the impact of SV on genes was checked by analyzing the intersection of the somatic SV and GRCh37 reference gene sets downloaded from the Ensemble website (https://grch37.ensembl.org/Homo_sapiens/Info/Annotation), using a threshold of 50% of the SV regions overlapping.

### 5.5 HBV Clonal Integration

First, we removed all reads irrelevant to the viral genomes, in which the input reads were aligned to the HBV genome using minimap2 (version 2.25) [50] with default parameters. The partially mapped reads were subjected to further analyses. Next, the sorted reads were realigned to the human genome (GRCh37) and the HBV genome to generate those possessing HBV integration sites. Finally, we utilized cuteSV (version 2.1) [51] to identify HBV integration sites via the following parameters: “–min_size 50 –max_size 50000000 –report_readid –min_support 1 –genotype –max_cluster_bias_INS 100 –diff_ratio_merging_INS 0.3 –max_cluster_bias_DEL 100 –diff_ratio_merging_DEL 0.3”. Breakends involving HBV sequences and human sequences were regarded as viral integration sites.

We also reconstructed HBV integration events based on multiple alignments of single nanopore reads. An HBV‐induced translocation can be associated with more than three distinct alignments in BAM files. The soft‐clips and hard‐clips of the reads involving HBV integration were recognized by “CIGAR” strings in each alignment.

### 5.6 Bulk‐RNA Sequencing Analysis

Total RNA was extracted from formalin‐fixed and paraffin‐embedded (FFPE) samples via an RNeasy FFPE Kit following the manufacturer's instructions. The RNA concentration was determined with a Qubit RNA HS Assay Kit and a Qubit 4.0 fluorometer, and the RNA integrity was determined via Qseq. Fifty nanograms of total RNA was used for reverse transcription and library construction. The libraries were sequenced on an Illumina NovaSeq 6000 platform using 150‐bp paired‐end reads. The RNA‐seq data were processed through HISAT2 and FeatureCounts packages with the GRCh37 human genome as a reference. DESeq2 R package was employed to calculate differential gene expression (|fold change| > 2, *p* < 0.05) among different age cohorts. For differential gene expression analysis, we used DESeq2 that incorporates internal normalization and statistical modeling. Gene set enrichment analysis and Kyoto Encyclopedia of Genes and Genomes (KEGG) pathway enrichment analysis and visualized the results were performed via GSEA (version: 4.3.2) and the ClusterProfiler package.

### 5.7 PCR Amplification and Sanger Sequencing

Genomic DNA was extracted from tumor tissues of candidate patients using TIANamp Genomic DNA Kit (Tiangen Biotech, Beijing, China). Specific gene fragments were amplified by PCR using gene‐specific primers (listed in Table S6). PCR reactions were carried out in a 50 µL reaction mixture containing < 500 ng of genomic DNA, 2 µL of each 10 µM primer, 25 µL 2× GoldStar Best MasterMix (Dye) (Cowin Biotech, Jiangsu, Beijing), and adding ddH_2_O to 50 µL. Thermal cycling conditions were as follows: initial denaturation at 95°C for 10 min, followed by 35 cycles of denaturation at 94°C for 15 s, annealing at 60°C for 30 s, extension at 72°C for 50 s, and a final extension at 72°C for 5 min. PCR products were verified by electrophoresis on a 1.5% agarose gel and visualized under UV light. PCR products of expected size were purified using the Gel Extraction Kit (Cowin Biotech) and subjected to Sanger sequencing (performed by Tsingke Biotechnology Co., Ltd, using the same primers as for amplification).

### 5.8 Statistical Analysis

The statistical tests are described in the figure legends. GraphPad Prism 8.0 was utilized for statistical analysis, and the data were displayed as the mean ± SD. The Student's *t*‐test or Wilcoxon rank‐sum test was used to compare two groups according to data distribution. The Fisher's exact test was used for comparison of frequency of HBV integration sites in different age groups. A difference is statistically significant if *p* < 0.05. Statistical *p*‐values are indicated in the figures as ****p < 0.0001, ***p <0.001, **p < 0.01, and *p < 0.05.

## Author Contributions

H.Z., Y.F.Z., X.Y.W., and J.B.L. conceived and directed the study. Z.W.W., Y.H.C., H.C.L., M.L., and B.L.Z analyzed and interpreted the data. J.M.Y., J.Q.C., X.Y.B., and J.J.Z. provided the study materials. J.G.Z., Z.Y.L., Z.H., J.M.L., and X.Y.L. provided essential biological resources and collected the clinical data. Z.W.L., Z.C.W., X.S.Z., Y.Y., Y.Q.D., Y.J.Y., and J.H.C. wrote the original drafts. All the authors approved the final manuscript and contributed to critical revisions to its intellectual context.

## Ethics Statement

The study was approved by the Ethics Committee of the National Cancer Center/Cancer Hospital, Chinese Academy of Medical Sciences, and Peking Union Medical College (NCC2023C‐620). All participants participated in the study with informed consent.

## Conflicts of Interest

The authors declare no conflicts of interest.

## Supporting information




**FIGURE S1**: SV distribution and validation of HBV integration sites near *DPRX* and *PIM3*. (A) Summary of gene/exon affected by somatic SVs. (B) HBV integration events located near the *DPRX* and *PIM3*. (C) Sanger sequencing results confirming HBV integration sites near *DPRX* and *PIM3*. (D) Agarose gel electrophoresis of PCR products corresponding to the validated integration sites.
**Table S1**: Clinical and pathological characterization of samples.
**Table S2**: Quality control metrics of whole‐genome sequencing data from 74 HCC patients.
**Table S3**: The number of different types of structural variation in 74 HCC patients.
**Table S4**: Chromosomal landscape of structural variation number.
**Table S5**: Distribution and proportion of repetitive elements across structural variation types.
**Table S6**: Sequences of primers used in this study.

## Data Availability

The raw sequencing data have been uploaded to the GSA‐Human database under accession code PRJCA027654 (https://ngdc.cncb.ac.cn/bioproject/browse/PRJCA027654).
