# Supplementary material for: Landscape of Somatic and Age‐Related Pathogenic Structural Variations in Hepatocellular Carcinoma Revealed by Long‐Read Sequencing
Source: MedComm (2020). 2026 Jan 13;7(1):e70570. doi: 10.1002/mco2.70570 (PMC12796842; doi:10.1002/mco2.70570)
Supplement: Supplementary file 1 — FIGURE S1: SV distribution and validation of HBV integration sites near DPRX and PIM3. (A) Summary of gene/exon affected by somatic SVs. (B) HBV integration events located near the DPRX and PIM3. (C) Sanger sequencing results confirming HBV integration sites near DPRX and PIM3. (D) Agarose gel electrophoresis of PCR products corresponding to the validated integration sites. Table S1: Clinical and pathological characterization of samples. Table S2: Quality control metrics of whole‐genome sequencing data from 74 HCC patients. Table S3: The number of different types of structural variation in 74 HCC patients. Table S4: Chromosomal landscape of structural variation number. Table S5: Distribution and proportion of repetitive elements across structural variation types. Table S6: Sequences of primers used in this study. [file MCO2-7-e70570-s001.docx]

**Landscape of Somatic and Age-Related Pathogenic Structural Variations in Hepatocellular Carcinoma Revealed by Long-Read Sequencing**

Running head: Somatic structural variations in HCC

Zhewen Wei^1,2,#^, Yinghao Cao^3,#^, Hongchao Liu^4,#^, Mei Liu^5,#^, Bolun Zhang^1,6,#^, Jianming Ying^7^, Jianqiang Cai^1,2^, Xinyu Bi^1,2^, Jianjun Zhao^1,2^, Jianguo Zhou^1,2^, Zhiyu Li^1,2^, Zhen Huang^1,2^, Jianmei Liu^1,2^, Xueyan Lv^1,2^, Zhiwen Luo^1,2^, Zhicheng Wei^1,2^, Xiaoshi Zhang^1,2^, Yi Yang^1,2^, Yiqiao Deng^1,2^, Yanjiang Yin^1,2^, Jinghua Chen^1,2^, Junbo Liang^8*^, Xiaoyue Wang^9*^, Yefan Zhang^1,2*^, Hong Zhao^1,2*^

^1^Department of Hepatobiliary Surgery, National Cancer Center/National Clinical Research Center for Cancer/Cancer Hospital, Chinese Academy of Medical Sciences and Peking Union Medical College, Beijing, China.

^2^Key Laboratory of Gene Editing Screening and R&D of Digestive System Tumor Drugs, Chinese Academy of Medical Sciences and Peking Union Medical College, Beijing, China.

^3^Center for Bioinformatics, Institute of Basic Medical Sciences Chinese Academy of Medical Sciences, School of Basic Medicine Peking Union Medical College, Beijing, China.

^4^Department of Laboratory Medicine, Peking University Third Hospital, Beijing, China.

^5^Laboratory of Cell and Molecular Biology & State Key Laboratory of Molecular Oncology, National Cancer Center/National Clinical Research Center for Cancer/Cancer Hospital, Chinese Academy of Medical Sciences and Peking Union Medical College, Beijing, China.

^6^Department of Hepatobiliary Surgery, Aerospace Center Hospital, Peking University Aerospace School of Clinical Medicine, Beijing, China.

^7^Department of Pathology, State Key Laboratory of Molecular Oncology, National Cancer Center/National Clinical Research Center for Cancer/Cancer Hospital, Chinese Academy of Medical Sciences and Peking Union Medical College, Beijing, China.

^8^State Key Laboratory of Common Mechanism Research for Major Diseases, Department of Biochemistry and Molecular Biology, Institute of Basic Medical Sciences Chinese Academy of Medical Sciences, School of Basic Medicine Peking Union Medical College, Beijing, China.

^9^State Key Laboratory of Common Mechanism Research for Major Diseases, Center for Bioinformatics, National Infrastructures for Translational Medicine, Institute of Clinical Medicine, Peking Union Medical College Hospital, Chinese Academy of Medical Science and Peking Union Medical College, Beijing, China.

^#^These authors contributed equally to this work.

*Correspondence: Junbo Liang ([liangjunbo@ibms.pumc.edu.cn](mailto:liangjunbo@ibms.pumc.edu.cn)); Xiaoyue Wang ([wxy@ibms.pumc.edu.cn](mailto:wxy@ibms.pumc.edu.cn)); Yefan Zhang ([yefanzhang@126.com](mailto:yefanzhang@126.com)); Hong Zhao ([zhaohong@cicams.ac.cn](mailto:zhaohong@cicams.ac.cn))


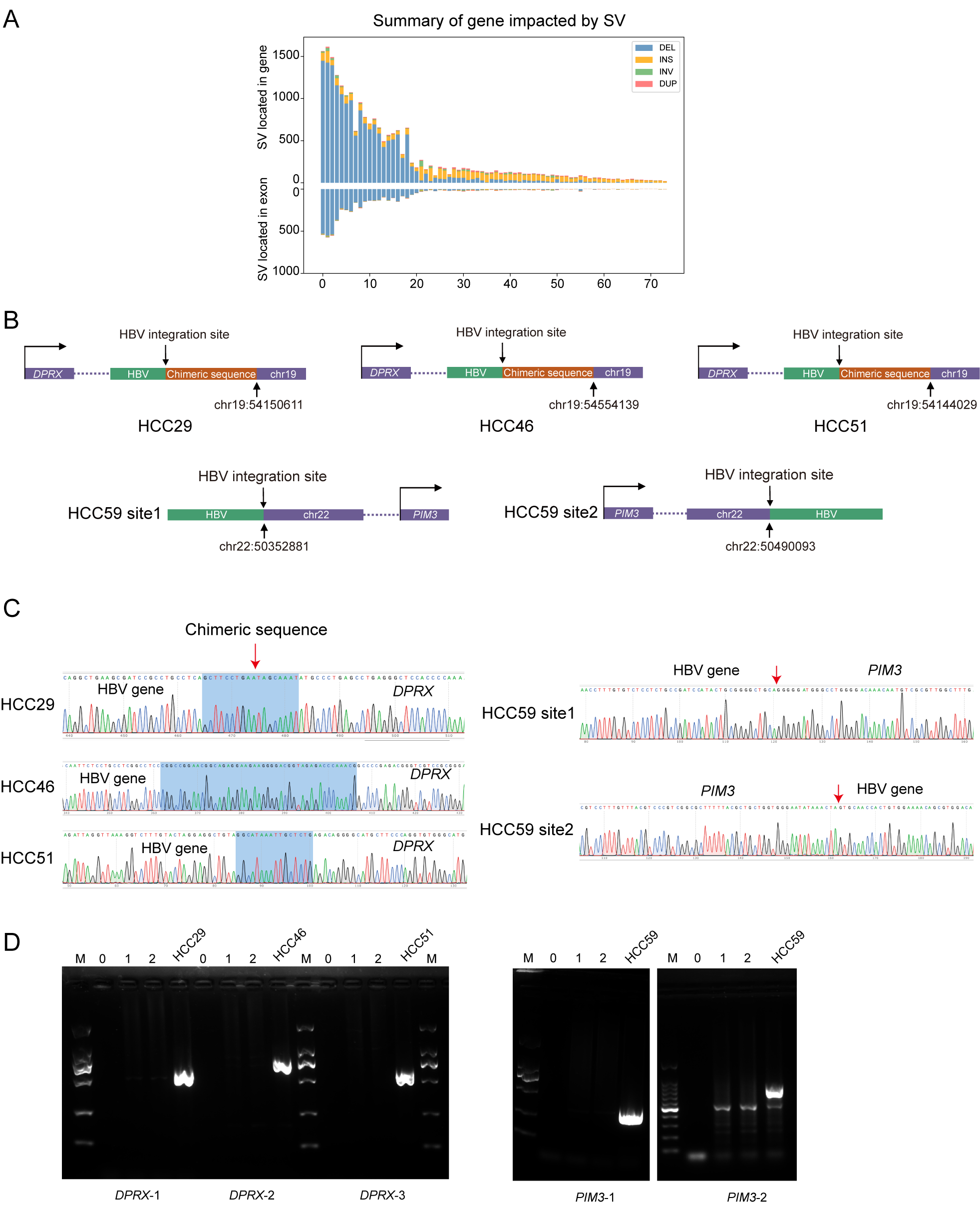


**FIGURE S1** SV distribution and validation of HBV integration sites near *DPRX* and *PIM3.* (A) Summary of gene/exon affected by somatic SVs. (B) HBV integration events located near the *DPRX* and *PIM3*. (C) Sanger sequencing results confirming HBV integration sites near *DPRX* and *PIM3.* (D) Agarose gel electrophoresis of PCR products corresponding to the validated integration sites.

**Table S1**. Clinical and pathological characterization of samples

| Sample | Sex | Age(years) | HBsAg | HCV-Ab | Hepatitis | Histological grade | Cirrhosis | BCLC | T stage | AFP (ng/mL) | Tumor diameter (mm) |  |
| --- | --- | --- | --- | --- | --- | --- | --- | --- | --- | --- | --- | --- |
| HCC01 | | Female | 26 | + | - | HBV | Ⅱ | N0 | B | 2 | >1210 | 30 |
| HCC02 | Male | 61 | - | + | HCV | Ⅱ | Yes | C | 2 | 848.7 | 25 |  |
| HCC03 | Male | 50 | - | + | HCV | Ⅱ | Yes | A | 2 | 4.45 | 25 |  |
| HCC04 | Male | 40 | + | - | HBV | Ⅲ | Yes | B | 2 | 2.6 | 25 |  |
| HCC05 | Female | 44 | + | - | HBV | Ⅱ | N0 | A | 2 | NA | 45 |  |
| HCC06 | Female | 40 | - | - | NBNC | Ⅱ | Yes | B | 2 | 161186 | 85 |  |
| HCC07 | Male | 61 | - | + | HCV | Ⅱ | Yes | A | 2 | 7.13 | 30 |  |
| HCC08 | Male | 66 | - | + | HCV | Ⅱ | Yes | A | 2 | 2.2 | 50 |  |
| HCC09 | Female | 58 | - | + | HCV | Ⅱ | Yes | C | 2 | >1210 | 90 |  |
| HCC10 | Female | 40 | - | + | HCV | Ⅱ | N0 | B | 2 | 86.77 | 65 |  |
| HCC11 | Male | 50 | - | + | HCV | Ⅱ | N0 | A | 2 | 5.17 | 43 |  |
| HCC12 | Male | 27 | - | - | NBNC | NA | NA | NA | NA | NA | NA |  |
| HCC13 | Male | 63 | - | + | HCV | Ⅱ | Yes | B | 2 | 5.48 | 40 |  |
| HCC14 | Male | 64 | - | - | NBNC | Ⅱ | N0 | B | 2 | >1210 | 90 |  |
| HCC15 | Male | 67 | - | - | NBNC | Ⅱ | Yes | A | 1 | 1.89 | 40 |  |
| HCC16 | Male | 61 | - | - | NBNC | Ⅱ | Yes | 0 | 2 | 2.09 | 13 |  |
| HCC17 | Male | 67 | - | + | HCV | Ⅰ | N0 | B | 1 | 5.01 | 80 |  |
| HCC18 | Male | 46 | - | - | NBNC | Ⅱ | Yes | A | 2 | 848 | 50 |  |
| HCC19 | Male | 67 | - | - | NBNC | Ⅱ | N0 | A | 1 | 2.43 | 33 |  |
| HCC20 | Male | 71 | - | - | NBNC | Ⅱ | N0 | B | 2 | 336 | 110 |  |
| HCC21 | Male | 49 | - | + | HCV | Ⅱ | Yes | A | 2 | 30.2 | 30 |  |
| HCC22 | Male | 33 | - | - | NBNC | Ⅲ | N0 | A | 2 | 1163 | 45 |  |
| HCC23 | Male | 34 | + | - | HBV | Ⅱ | N0 | B | 2 | 6088 | 20 |  |
| HCC24 | Male | 32 | + | - | HBV | Ⅱ | N0 | B | 2 | 19222 | 40 |  |
| HCC25 | Male | 22 | + | - | HBV | Ⅱ | Yes | C | 4 | 1275 | 22 |  |
| HCC26 | Male | 30 | + | - | HBV | Ⅱ | Yes | A | 2 | NA | 100 |  |
| HCC27 | Male | 32 | + | - | HBV | Ⅱ | Yes | A | 2 | 69.93 | 130 |  |
| HCC28 | Female | 31 | + | - | HBV | Ⅱ | N0 | B | 2 | 1356 | 65 |  |
| HCC29 | Male | 16 | + | - | HBV | Ⅱ | Yes | C | 2 | >1000 | 125 |  |
| HCC30 | Male | 25 | - | - | NBNC | Ⅰ | N0 | B | 1 | 3.1 | 120 |  |
| HCC31 | Male | 29 | + | - | HBV | Ⅱ | Yes | B | 2 | >1210 | 140 |  |
| HCC32 | Male | 31 | + | - | HBV | Ⅱ | N0 | A | 2 | 106 | 115 |  |
| HCC33 | Male | 32 | + | - | HBV | Ⅱ | N0 | B | 2 | 1.85 | 28 |  |
| HCC34 | Male | 34 | + | - | HBV | Ⅱ | Yes | B | 2 | >1210 | 70 |  |
| HCC35 | Male | 25 | - | - | NBNC | Ⅱ | N0 | B | 2 | 2.22 | 62 |  |
| HCC36 | Male | 24 | + | - | HBV | Ⅱ | Yes | B | 2 | 104.9 | 105 |  |
| HCC37 | Male | 28 | + | - | HBV | Ⅱ | N0 | B | 2 | >1210 | 47 |  |
| HCC38 | Male | 35 | + | - | HBV | Ⅱ | Yes | A | 2 | 1334 | 57 |  |
| HCC39 | Male | 23 | + | - | HBV | Ⅱ | Yes | B | 2 | >1210 | 140 |  |
| HCC40 | Male | 21 | + | - | HBV | Ⅱ | Yes | B | 1 | 4018 | 80 |  |
| HCC41 | Male | 41 | + | - | HBV | Ⅱ | N0 | A | 2 | 4.06 | 90 |  |
| HCC42 | Male | 29 | + | - | HBV | Ⅱ | N0 | A | 3 | 793.6 | 60 |  |
| HCC43 | Male | 31 | + | - | HBV | Ⅱ | Yes | B | 1 | 2.45 | 40 |  |
| HCC44 | Male | 30 | + | - | HBV | Ⅱ | Yes | A | 2 | 5.15 | 65 |  |
| HCC45 | Male | 66 | + | - | HBV | Ⅲ | Yes | 0 | 1 | 523.4 | 47 |  |
| HCC46 | Male | 25 | + | - | HBV | Ⅱ | N0 | B | 2 | >24200 | 30 |  |
| HCC47 | Male | 30 | + | - | HBV | Ⅱ | Yes | A | 2 | 9.94 | 120 |  |
| HCC48 | Male | 63 | + | - | HBV | Ⅱ | Yes | B | 1 | 20.3 | 110 |  |
| HCC49 | Female | 29 | + | - | HBV | Ⅲ | Yes | B | 2 | 3.11 | 32 |  |
| HCC50 | Female | 30 | + | - | HBV | Ⅱ | N0 | A | 4 | 1231 | 25 |  |
| HCC51 | Female | 34 | + | - | HBV | Ⅱ | N0 | B | 1 | 285829 | 25 |  |
| HCC52 | Female | 29 | + | - | HBV | Ⅱ | N0 | A | 2 | 4895 | 58 |  |
| HCC53 | Male | 50 | - | + | HCV | Ⅱ | Yes | A | 1 | 9.67 | 25 |  |
| HCC54 | Female | 74 | - | - | NBNC | Ⅱ | N0 | B | 4 | 16.64 | 120 |  |
| HCC55 | Female | 65 | + | - | HBV | Ⅱ | Yes | A | 2 | 2.48 | 90 |  |
| HCC56 | Male | 53 | - | - | NBNC | Ⅰ | Yes | A | 1 | 6.52 | 42 |  |
| HCC57 | Male | 57 | - | - | NBNC | Ⅱ | N0 | A | 1 | 2.07 | 45 |  |
| HCC58 | Female | 72 | - | + | HCV | Ⅱ | N0 | B | 1 | 3.15 | 52 |  |
| HCC59 | Male | 22 | + | - | HBV | Ⅱ | Yes | B | 3 | >1210 | 100 |  |
| HCC60 | Female | 69 | - | + | HCV | Ⅱ | N0 | A | 1 | 2.14 | 35 |  |
| HCC61 | Male | 70 | + | - | HBV | Ⅱ | N0 | B | 2 | 22.72 | 50 |  |
| HCC62 | Male | 61 | - | - | NBNC | Ⅱ | Yes | B | 2 | 2956 | 60 |  |
| HCC63 | Male | 73 | - | + | HCV | Ⅰ | N0 | A | 2 | 4.44 | 38 |  |
| HCC64 | Male | 67 | - | - | NBNC | Ⅱ | Yes | A | 1 | 7.68 | 48 |  |
| HCC65 | Male | 35 | + | - | HBV | Ⅱ | Yes | B | 2 | 14181 | 70 |  |
| HCC66 | Male | 55 | - | - | NBNC | Ⅲ | Yes | A | 2 | 613.2 | 45 |  |
| HCC67 | Male | 48 | + | - | HBV | Ⅱ | Yes | A | 2 | 14.47 | 50 |  |
| HCC68 | Male | 49 | + | - | HBV | Ⅲ | Yes | B | 2 | 164.1 | 30 |  |
| HCC69 | Male | 69 | + | - | HBV | Ⅱ | Yes | A | 2 | 17.15 | 35 |  |
| HCC70 | Male | 60 | + | - | HBV | Ⅱ | Yes | B | 1 | 3.1 | 85 |  |
| HCC71 | Male | 53 | + | - | HBV | Ⅲ | Yes | A | 1 | 1.93 | 30 |  |
| HCC72 | Male | 68 | + | - | HBV | Ⅱ | Yes | B | 2 | 5.73 | 110 |  |
| HCC73 | Male | 58 | + | - | HBV | Ⅲ | Yes | A | 1 | 403 | 20 |  |
| HCC74 | Female | 78 | + | - | HBV | Ⅲ | Yes | B | 2 | 15765 | 52 |  |

**Table S2.** Quality control metrics of whole-genome sequencing data from 74 HCC patients.

| **Sample** | **Total base (Gbp)** | **Number of reads** | **Average read length (bp)** | **Maximum read length (bp)** | **Number of mapped reads** | **Number of unmapped reads** | **Mapping rate** | **Total length of mapped bases (Gbp)** | **Proportion of mapped based** |
| --- | --- | --- | --- | --- | --- | --- | --- | --- | --- |
| HCC01_N | 43.3 | 2,821,288 | 15,362 | 178,895 | 2,689,893 | 131,395 | 95.3% | 41.7 | 96.2% |
| HCC01_T | 45.8 | 5,540,990 | 8,264 | 154,304 | 5,288,300 | 252,690 | 95.4% | 44.2 | 96.5% |
| HCC02_N | 43.2 | 3,359,202 | 12,848 | 168,932 | 3,218,035 | 141,167 | 95.8% | 41.8 | 96.8% |
| HCC02_T | 40.1 | 2,833,020 | 14,146 | 177,914 | 2,720,922 | 112,098 | 96.0% | 38.8 | 96.8% |
| HCC03_N | 39.4 | 2,081,499 | 18,930 | 178,773 | 2,004,749 | 76,750 | 96.3% | 38.2 | 97.0% |
| HCC03_T | 28.2 | 2,010,069 | 14,034 | 187,592 | 1,919,804 | 90,265 | 95.5% | 27.2 | 96.5% |
| HCC04_N | 48.2 | 6,887,186 | 6,993 | 152,803 | 6,473,122 | 414,064 | 94.0% | 46.4 | 96.4% |
| HCC04_T | 47.9 | 14,436,058 | 3,315 | 143,792 | 13,261,999 | 1,174,059 | 91.9% | 45.4 | 94.9% |
| HCC05_N | 49.0 | 4,579,369 | 10,693 | 698,730 | 4,338,707 | 240,662 | 94.7% | 47.3 | 96.7% |
| HCC05_T | 46.5 | 13,278,362 | 3,498 | 149,982 | 12,284,465 | 993,897 | 92.5% | 44.1 | 94.9% |
| HCC06_N | 49.8 | 9,255,969 | 5,375 | 116,127 | 8,793,839 | 462,130 | 95.0% | 47.9 | 96.2% |
| HCC06_T | 52.6 | 13,215,306 | 3,982 | 296,788 | 12,311,310 | 903,996 | 93.2% | 50.2 | 95.4% |
| HCC07_N | 52.6 | 3,685,733 | 14,260 | 178,521 | 3,542,458 | 143,275 | 96.1% | 50.8 | 96.7% |
| HCC07_T | 49.5 | 9,780,139 | 5,061 | 98,457 | 9,326,044 | 454,095 | 95.4% | 47.9 | 96.7% |
| HCC08_N | 49.9 | 8,093,976 | 6,169 | 257,651 | 7,415,054 | 678,922 | 91.6% | 47.8 | 95.7% |
| HCC08_T | 51.8 | 3,508,470 | 14,751 | 217,889 | 3,365,729 | 142,741 | 95.9% | 50.2 | 96.9% |
| HCC09_N | 44.0 | 2,255,752 | 19,513 | 211,878 | 2,144,878 | 110,874 | 95.1% | 42.2 | 95.9% |
| HCC09_T | 58.4 | 11,772,002 | 4,959 | 155,944 | 11,184,700 | 587,302 | 95.0% | 55.9 | 95.7% |
| HCC10_N | 44.4 | 7,351,537 | 6,043 | 140,132 | 6,955,511 | 396,026 | 94.6% | 42.5 | 95.7% |
| HCC10_T | 32.9 | 1,774,943 | 18,542 | 192,036 | 1,692,953 | 81,990 | 95.4% | 31.7 | 96.2% |
| HCC11_N | 46.9 | 6,067,959 | 7,721 | 114,127 | 5,833,856 | 234,103 | 96.1% | 45.5 | 97.1% |
| HCC11_T | 59.6 | 16,964,039 | 3,510 | 132,458 | 16,273,868 | 690,171 | 95.9% | 57.6 | 96.6% |
| HCC12_N | 58.1 | 15,734,257 | 3,691 | 893,692 | 14,689,781 | 1,044,476 | 93.4% | 55.6 | 95.8% |
| HCC12_T | 51.8 | 16,969,133 | 3,054 | 76,014 | 15,963,209 | 1,005,924 | 94.1% | 49.6 | 95.6% |
| HCC13_N | 45.6 | 8,350,140 | 5,457 | 118,766 | 7,877,670 | 472,470 | 94.3% | 43.8 | 96.0% |
| HCC13_T | 54.1 | 8,086,607 | 6,689 | 191,458 | 7,638,456 | 448,151 | 94.5% | 51.9 | 95.9% |
| HCC14_N | 53.0 | 19,247,915 | 2,755 | 216,609 | 18,193,297 | 1,054,618 | 94.5% | 50.7 | 95.6% |
| HCC14_T | 43.5 | 8,421,071 | 5,170 | 98,885 | 8,093,781 | 327,290 | 96.1% | 42.3 | 97.2% |
| HCC15_N | 44.0 | 2,679,603 | 16,421 | 221,755 | 2,581,529 | 98,074 | 96.3% | 42.6 | 96.7% |
| HCC15_T | 36.3 | 4,595,797 | 7,908 | 122,775 | 4,386,723 | 209,074 | 95.5% | 35.1 | 96.6% |
| HCC16_N | 48.3 | 4,166,069 | 11,590 | 142,983 | 4,029,286 | 136,783 | 96.7% | 47.0 | 97.4% |
| HCC16_T | 41.4 | 2,890,226 | 14,309 | 157,008 | 2,805,227 | 84,999 | 97.1% | 40.3 | 97.6% |
| HCC17_N | 51.3 | 11,866,593 | 4,319 | 182,654 | 11,108,840 | 757,753 | 93.6% | 48.9 | 95.4% |
| HCC17_T | 38.9 | 5,436,127 | 7,147 | 214,164 | 5,087,296 | 348,831 | 93.6% | 37.3 | 96.1% |
| HCC18_N | 41.1 | 2,107,170 | 19,528 | 205,494 | 2,026,443 | 80,727 | 96.2% | 39.8 | 96.8% |
| HCC18_T | 44.9 | 1,923,217 | 23,347 | 165,156 | 1,852,709 | 70,508 | 96.3% | 43.5 | 96.8% |
| HCC19_N | 46.6 | 5,639,450 | 8,270 | 105,613 | 5,483,704 | 155,746 | 97.2% | 45.8 | 98.1% |
| HCC19_T | 46.6 | 4,512,059 | 10,338 | 139,784 | 4,344,244 | 167,815 | 96.3% | 45.3 | 97.2% |
| HCC20_N | 49.7 | 8,980,095 | 5,533 | 144,572 | 8,553,643 | 426,452 | 95.3% | 47.9 | 96.4% |
| HCC20_T | 43.5 | 8,737,775 | 4,977 | 180,795 | 8,406,334 | 331,441 | 96.2% | 42.3 | 97.2% |
| HCC21_N | 47.8 | 20,984,885 | 2,276 | 91,815 | 19,012,834 | 1,972,051 | 90.6% | 44.5 | 93.2% |
| HCC21_T | 43.2 | 2,426,260 | 17,809 | 179,245 | 2,347,018 | 79,242 | 96.7% | 42.1 | 97.4% |
| HCC22_N | 78.6 | 4,896,170 | 16,053 | 163,275 | 4,742,013 | 154,157 | 96.9% | 76.3 | 97.1% |
| HCC22_T | 57.6 | 10,683,088 | 5,388 | 194,089 | 10,175,386 | 507,702 | 95.2% | 55.4 | 96.2% |
| HCC23_N | 28.2 | 3,256,312 | 8,651 | 250,945 | 3,125,679 | 130,633 | 96.0% | 27.4 | 97.1% |
| HCC23_T | 55.4 | 13,223,401 | 4,192 | 231,404 | 12,420,672 | 802,729 | 93.9% | 53.0 | 95.7% |
| HCC24_N | 33.7 | 7,124,797 | 4,735 | 119,498 | 6,775,079 | 349,718 | 95.1% | 32.4 | 96.0% |
| HCC24_T | 56.9 | 21,239,120 | 2,678 | 72,232 | 19,373,723 | 1,865,397 | 91.2% | 53.2 | 93.6% |
| HCC25_N | 49.3 | 4,778,080 | 10,315 | 171,226 | 4,594,356 | 183,724 | 96.2% | 47.7 | 96.9% |
| HCC25_T | 32.1 | 1,481,139 | 21,672 | 194,705 | 1,420,303 | 60,836 | 95.9% | 31.0 | 96.6% |
| HCC26_N | 50.0 | 4,541,046 | 11,009 | 217,791 | 4,316,183 | 224,863 | 95.0% | 48.1 | 96.3% |
| HCC26_T | 41.1 | 2,369,105 | 17,361 | 176,996 | 2,267,025 | 102,080 | 95.7% | 39.7 | 96.5% |
| HCC27_N | 49.1 | 4,356,067 | 11,268 | 140,177 | 4,182,259 | 173,808 | 96.0% | 47.5 | 96.7% |
| HCC27_T | 51.8 | 4,541,648 | 11,410 | 137,709 | 4,367,288 | 174,360 | 96.2% | 50.3 | 97.1% |
| HCC28_N | 44.9 | 4,086,591 | 10,993 | 164,673 | 3,902,206 | 184,385 | 95.5% | 43.4 | 96.7% |
| HCC28_T | 47.0 | 4,221,713 | 11,135 | 154,619 | 4,033,120 | 188,593 | 95.5% | 45.6 | 97.0% |
| HCC29_N | 44.5 | 6,879,193 | 6,468 | 130,341 | 6,599,626 | 279,567 | 95.9% | 43.3 | 97.3% |
| HCC29_T | 39.2 | 5,743,063 | 6,825 | 114,897 | 5,480,601 | 262,462 | 95.4% | 37.8 | 96.5% |
| HCC30_N | 72.5 | 8,490,596 | 8,537 | 121,295 | 8,162,791 | 327,805 | 96.1% | 70.2 | 96.8% |
| HCC30_T | 41.8 | 4,158,838 | 10,059 | 144,467 | 3,990,910 | 167,928 | 96.0% | 40.5 | 96.9% |
| HCC31_N | 46.8 | 1,927,740 | 24,261 | 1,068,744 | 1,832,650 | 95,090 | 95.1% | 44.7 | 95.5% |
| HCC31_T | 46.4 | 2,971,249 | 15,612 | 200,230 | 2,833,455 | 137,794 | 95.4% | 44.6 | 96.2% |
| HCC32_N | 43.5 | 3,683,090 | 11,809 | 118,742 | 3,572,311 | 110,779 | 97.0% | 42.3 | 97.3% |
| HCC32_T | 42.3 | 2,848,619 | 14,861 | 184,015 | 2,765,918 | 82,701 | 97.1% | 41.3 | 97.5% |
| HCC33_N | 72.4 | 8,617,214 | 8,403 | 209,760 | 8,360,067 | 257,147 | 97.0% | 70.5 | 97.3% |
| HCC33_T | 44.5 | 6,963,690 | 6,390 | 90,152 | 6,569,650 | 394,040 | 94.3% | 42.7 | 95.9% |
| HCC34_N | 32.9 | 1,422,523 | 23,111 | 198,434 | 1,361,090 | 61,433 | 95.7% | 31.6 | 96.1% |
| HCC34_T | 47.0 | 3,182,523 | 14,776 | 177,624 | 3,035,793 | 146,730 | 95.4% | 45.3 | 96.3% |
| HCC35_N | 47.8 | 3,357,156 | 14,249 | 182,584 | 3,247,342 | 109,814 | 96.7% | 46.5 | 97.1% |
| HCC35_T | 57.1 | 3,668,423 | 15,570 | 666,769 | 3,547,607 | 120,816 | 96.7% | 55.6 | 97.3% |
| HCC36_N | 41.4 | 8,479,944 | 4,882 | 213,635 | 7,799,331 | 680,613 | 92.0% | 39.5 | 95.3% |
| HCC36_T | 24.8 | 1,134,760 | 21,827 | 202,772 | 1,089,183 | 45,577 | 96.0% | 23.9 | 96.5% |
| HCC37_N | 57.2 | 6,065,736 | 9,435 | 171,597 | 5,797,384 | 268,352 | 95.6% | 55.4 | 96.8% |
| HCC37_T | 50.8 | 6,815,444 | 7,451 | 148,157 | 6,486,571 | 328,873 | 95.2% | 49.1 | 96.6% |
| HCC38_N | 49.6 | 15,487,693 | 3,203 | 173,419 | 14,642,246 | 845,447 | 94.5% | 47.3 | 95.4% |
| HCC38_T | 46.1 | 7,958,083 | 5,792 | 132,116 | 7,563,817 | 394,266 | 95.0% | 44.5 | 96.4% |
| HCC39_N | 49.0 | 10,078,910 | 4,864 | 242,378 | 9,357,286 | 721,624 | 92.8% | 46.8 | 95.4% |
| HCC39_T | 58.4 | 6,587,160 | 8,865 | 239,724 | 6,248,974 | 338,186 | 94.9% | 56.2 | 96.2% |
| HCC40_N | 41.0 | 4,267,768 | 9,606 | 129,126 | 4,085,698 | 182,070 | 95.7% | 39.7 | 96.9% |
| HCC40_T | 46.4 | 16,399,549 | 2,827 | 78,480 | 14,848,141 | 1,551,408 | 90.5% | 43.0 | 92.8% |
| HCC41_N | 31.8 | 2,398,430 | 13,268 | 160,807 | 2,320,753 | 77,677 | 96.8% | 31.0 | 97.4% |
| HCC41_T | 40.8 | 7,405,156 | 5,507 | 197,940 | 6,981,816 | 423,340 | 94.3% | 39.0 | 95.7% |
| HCC42_N | 53.7 | 4,023,524 | 13,352 | 159,640 | 3,846,636 | 176,888 | 95.6% | 52.0 | 96.9% |
| HCC42_T | 44.9 | 2,311,914 | 19,419 | 295,578 | 2,208,955 | 102,959 | 95.5% | 43.1 | 96.1% |
| HCC43_N | 48.1 | 3,950,607 | 12,186 | 173,356 | 3,743,181 | 207,426 | 94.7% | 46.6 | 96.8% |
| HCC43_T | 50.6 | 11,403,987 | 4,441 | 277,712 | 10,613,025 | 790,962 | 93.1% | 48.7 | 96.1% |
| HCC44_N | 62.6 | 37,792,825 | 1,655 | 114,112 | 34,279,146 | 3,513,679 | 90.7% | 57.7 | 92.2% |
| HCC44_T | 48.6 | 7,901,944 | 6,151 | 144,670 | 7,593,142 | 308,802 | 96.1% | 47.4 | 97.5% |
| HCC45_N | 52.7 | 12,839,214 | 4,106 | 119,069 | 12,194,089 | 645,125 | 95.0% | 50.7 | 96.1% |
| HCC45_T | 50.7 | 4,323,897 | 11,735 | 825,215 | 4,201,719 | 122,178 | 97.2% | 49.5 | 97.6% |
| HCC46_N | 51.2 | 8,426,878 | 6,072 | 225,149 | 8,106,756 | 320,122 | 96.2% | 49.6 | 97.0% |
| HCC46_T | 56.3 | 6,529,835 | 8,615 | 144,784 | 6,211,178 | 318,657 | 95.1% | 54.5 | 96.9% |
| HCC47_N | 44.5 | 2,154,017 | 20,673 | 168,415 | 2,077,598 | 76,419 | 96.5% | 43.2 | 97.0% |
| HCC47_T | 42.4 | 2,007,602 | 21,123 | 211,773 | 1,926,854 | 80,748 | 96.0% | 41.0 | 96.6% |
| HCC48_N | 63.6 | 4,473,751 | 14,207 | 180,274 | 4,341,503 | 132,248 | 97.0% | 62.1 | 97.7% |
| HCC48_T | 47.7 | 11,870,518 | 4,020 | 112,393 | 11,232,951 | 637,567 | 94.6% | 45.7 | 95.9% |
| HCC49_N | 47.4 | 2,349,390 | 20,168 | 176,817 | 2,276,461 | 72,929 | 96.9% | 46.1 | 97.4% |
| HCC49_T | 44.6 | 5,173,297 | 8,614 | 196,360 | 5,031,976 | 141,321 | 97.3% | 43.6 | 97.9% |
| HCC50_N | 57.2 | 5,716,029 | 10,008 | 184,483 | 5,537,411 | 178,618 | 96.9% | 55.6 | 97.3% |
| HCC50_T | 72.8 | 15,408,736 | 4,721 | 152,561 | 14,809,546 | 599,190 | 96.1% | 70.4 | 96.7% |
| HCC51_N | 48.4 | 4,921,690 | 9,830 | 644,813 | 4,720,413 | 201,277 | 95.9% | 47.0 | 97.2% |
| HCC51_T | 28.9 | 2,691,291 | 10,749 | 150,713 | 2,579,241 | 112,050 | 95.8% | 28.0 | 96.7% |
| HCC52_N | 47.7 | 3,695,295 | 12,900 | 263,154 | 3,558,600 | 136,695 | 96.3% | 46.2 | 97.0% |
| HCC52_T | 50.5 | 5,610,885 | 8,993 | 165,916 | 5,370,409 | 240,476 | 95.7% | 48.9 | 96.9% |
| HCC53_N | 53.6 | 3,718,129 | 14,405 | 158,880 | 3,607,471 | 110,658 | 97.0% | 52.3 | 97.6% |
| HCC53_T | 57.0 | 6,336,286 | 8,994 | 361,069 | 6,103,218 | 233,068 | 96.3% | 55.2 | 96.9% |
| HCC54_N | 52.8 | 4,571,494 | 11,552 | 103,633 | 4,423,650 | 147,844 | 96.8% | 51.3 | 97.1% |
| HCC54_T | 56.2 | 13,721,383 | 4,096 | 141,842 | 12,909,445 | 811,938 | 94.1% | 53.8 | 95.7% |
| HCC55_N | 50.1 | 4,548,530 | 11,012 | 154,000 | 4,393,623 | 154,907 | 96.6% | 48.7 | 97.3% |
| HCC55_T | 57.9 | 15,754,545 | 3,673 | 111,076 | 14,665,944 | 1,088,601 | 93.1% | 55.0 | 95.0% |
| HCC56_N | 42.1 | 5,826,063 | 7,227 | 97,417 | 5,552,727 | 273,336 | 95.3% | 40.6 | 96.5% |
| HCC56_T | 64.7 | 4,697,505 | 13,763 | 232,515 | 4,558,550 | 138,955 | 97.0% | 62.9 | 97.3% |
| HCC57_N | 48.3 | 10,163,868 | 4,753 | 188,342 | 9,604,107 | 559,761 | 94.5% | 46.3 | 95.9% |
| HCC57_T | 77.0 | 6,223,033 | 12,376 | 164,026 | 6,032,780 | 190,253 | 96.9% | 74.8 | 97.1% |
| HCC58_N | 71.8 | 6,309,275 | 11,377 | 114,947 | 6,084,409 | 224,866 | 96.4% | 69.7 | 97.1% |
| HCC58_T | 48.8 | 16,569,924 | 2,944 | 106,805 | 15,318,727 | 1,251,197 | 92.4% | 46.2 | 94.6% |
| HCC59_N | 47.1 | 6,569,400 | 7,175 | 177,709 | 6,246,115 | 323,285 | 95.1% | 45.3 | 96.1% |
| HCC59_T | 52.0 | 5,762,792 | 9,026 | 188,388 | 5,453,099 | 309,693 | 94.6% | 49.9 | 95.9% |
| HCC60_N | 46.1 | 3,038,692 | 15,162 | 142,727 | 2,942,497 | 96,195 | 96.8% | 44.8 | 97.2% |
| HCC60_T | 42.6 | 18,541,680 | 2,295 | 64,665 | 16,838,394 | 1,703,286 | 90.8% | 39.7 | 93.4% |
| HCC61_N | 28.7 | 5,099,549 | 5,626 | 97,728 | 4,859,676 | 239,873 | 95.3% | 27.7 | 96.6% |
| HCC61_T | 43.1 | 8,861,357 | 4,863 | 181,616 | 8,320,079 | 541,278 | 93.9% | 41.4 | 96.0% |
| HCC62_N | 46.5 | 13,253,465 | 3,510 | 110,494 | 12,345,347 | 908,118 | 93.1% | 44.4 | 95.4% |
| HCC62_T | 33.0 | 8,554,011 | 3,852 | 155,214 | 7,662,909 | 891,102 | 89.6% | 31.6 | 96.0% |
| HCC63_N | 84.4 | 14,348,703 | 5,884 | 132,138 | 13,570,603 | 778,100 | 94.6% | 80.6 | 95.4% |
| HCC63_T | 58.4 | 8,558,874 | 6,817 | 101,126 | 8,175,849 | 383,025 | 95.5% | 56.7 | 97.2% |
| HCC64_N | 49.0 | 5,217,243 | 9,382 | 116,011 | 5,038,632 | 178,611 | 96.6% | 47.7 | 97.4% |
| HCC64_T | 48.9 | 3,143,937 | 15,547 | 156,966 | 2,999,902 | 144,035 | 95.4% | 47.1 | 96.4% |
| HCC65_N | 46.6 | 9,911,502 | 4,699 | 371,157 | 9,512,792 | 398,710 | 96.0% | 45.3 | 97.3% |
| HCC65_T | 41.5 | 3,395,216 | 12,209 | 183,428 | 3,306,207 | 89,009 | 97.4% | 40.8 | 98.3% |
| HCC66_N | 36.6 | 8,982,116 | 4,079 | 185,028 | 8,467,551 | 514,565 | 94.3% | 35.0 | 95.6% |
| HCC66_T | 73.3 | 8,996,364 | 8,151 | 226,378 | 8,694,953 | 301,411 | 96.6% | 71.3 | 97.2% |
| HCC67_N | 73.3 | 6,871,595 | 10,667 | 156,749 | 6,644,990 | 226,605 | 96.7% | 71.5 | 97.5% |
| HCC67_T | 38.1 | 5,004,516 | 7,615 | 163,589 | 4,702,059 | 302,457 | 94.0% | 36.8 | 96.7% |
| HCC68_N | 50.6 | 5,224,308 | 9,680 | 207,410 | 5,018,196 | 206,112 | 96.1% | 49.1 | 97.2% |
| HCC68_T | 47.5 | 6,549,791 | 7,258 | 185,437 | 6,165,168 | 384,623 | 94.1% | 45.9 | 96.5% |
| HCC69_N | 53.3 | 5,684,765 | 9,376 | 250,437 | 5,300,141 | 384,624 | 93.2% | 51.3 | 96.3% |
| HCC69_T | 47.3 | 5,701,754 | 8,292 | 257,655 | 5,297,088 | 404,666 | 92.9% | 45.4 | 96.0% |
| HCC70_N | 42.6 | 23,659,131 | 1,802 | 308,111 | 20,789,098 | 2,870,033 | 87.9% | 40.0 | 93.8% |
| HCC70_T | 45.7 | 3,903,147 | 11,712 | 363,624 | 3,763,621 | 139,526 | 96.4% | 44.4 | 97.2% |
| HCC71_N | 45.4 | 5,445,665 | 8,341 | 258,601 | 5,104,122 | 341,543 | 93.7% | 43.7 | 96.3% |
| HCC71_T | 43.0 | 8,384,236 | 5,133 | 335,920 | 7,919,563 | 464,673 | 94.5% | 41.3 | 96.0% |
| HCC72_N | 42.9 | 9,078,098 | 4,720 | 275,795 | 8,496,071 | 582,027 | 93.6% | 40.8 | 95.3% |
| HCC72_T | 51.4 | 6,823,342 | 7,532 | 305,964 | 6,308,835 | 514,507 | 92.5% | 49.5 | 96.3% |
| HCC73_N | 57.3 | 4,221,243 | 13,576 | 218,089 | 3,951,672 | 269,571 | 93.6% | 55.3 | 96.5% |
| HCC73_T | 50.0 | 15,310,018 | 3,268 | 123,201 | 14,121,080 | 1,188,938 | 92.2% | 47.0 | 94.0% |
| HCC74_N | 51.9 | 12,604,206 | 4,118 | 147,210 | 11,922,843 | 681,363 | 94.6% | 49.7 | 95.8% |
| HCC74_T | 57.1 | 4,320,876 | 13,214 | 125,827 | 4,203,981 | 116,895 | 97.3% | 55.7 | 97.6% |

**Table S3.** The number of different types of structural variation in 74 HCC patients.

| ID | Hepatitis | Sex | DEL | INS | INV | DUP | Total |
| --- | --- | --- | --- | --- | --- | --- | --- |
| HCC49 | HBV | Female | 13944 | 239 | 24 | 21 | 14228 |
| HCC44 | HBV | Male | 13006 | 305 | 67 | 24 | 13402 |
| HCC41 | HBV | Male | 12970 | 145 | 32 | 18 | 13165 |
| HCC42 | HBV | Male | 8732 | 186 | 76 | 12 | 9006 |
| HCC46 | HBV | Female | 5291 | 200 | 7 | 14 | 5512 |
| HCC38 | HBV | Female | 4716 | 186 | 8 | 7 | 4917 |
| HCC27 | HBV | Male | 4712 | 176 | 12 | 10 | 4910 |
| HCC51 | HBV | Male | 4217 | 90 | 4 | 11 | 4322 |
| HCC31 | HBV | Female | 4073 | 182 | 10 | 15 | 4280 |
| HCC34 | HBV | Female | 3692 | 179 | 1 | 18 | 3890 |
| HCC25 | HBV | Male | 2890 | 137 | 6 | 8 | 3041 |
| HCC22 | NBNC | Male | 2732 | 121 | 6 | 21 | 2880 |
| HCC01 | HBV | Male | 2417 | 140 | 14 | 8 | 2579 |
| HCC28 | HBV | Male | 2251 | 165 | 0 | 23 | 2439 |
| HCC39 | HBV | Male | 2263 | 127 | 11 | 13 | 2414 |
| HCC29 | HBV | Male | 2131 | 147 | 6 | 14 | 2298 |
| HCC52 | HBV | Male | 2175 | 109 | 8 | 5 | 2297 |
| HCC30 | NBNC | Male | 2008 | 129 | 0 | 11 | 2148 |
| HCC43 | HBV | Male | 1934 | 170 | 8 | 13 | 2125 |
| HCC37 | HBV | Male | 1820 | 114 | 1 | 10 | 1945 |
| HCC26 | HBV | Male | 1008 | 95 | 2 | 7 | 1112 |
| HCC48 | HBV | Male | 58 | 400 | 122 | 28 | 608 |
| HCC59 | HBV | Male | 396 | 105 | 1 | 13 | 515 |
| HCC57 | NBNC | Male | 48 | 360 | 36 | 20 | 464 |
| HCC36 | HBV | Male | 385 | 61 | 0 | 1 | 447 |
| HCC74 | HBV | Male | 92 | 270 | 11 | 30 | 403 |
| HCC69 | HBV | Male | 81 | 251 | 35 | 29 | 396 |
| HCC47 | HBV | Female | 311 | 72 | 0 | 5 | 388 |
| HCC70 | HBV | Male | 120 | 231 | 7 | 29 | 387 |
| HCC68 | HBV | Male | 116 | 200 | 15 | 25 | 356 |
| HCC06 | NBNC | Male | 59 | 228 | 19 | 19 | 325 |
| HCC73 | HBV | Male | 109 | 180 | 17 | 19 | 325 |
| HCC05 | HBV | Male | 74 | 208 | 3 | 37 | 322 |
| HCC08 | HCV | Male | 113 | 161 | 4 | 29 | 307 |
| HCC62 | NBNC | Male | 41 | 220 | 31 | 14 | 306 |
| HCC40 | HBV | Male | 16 | 223 | 33 | 25 | 297 |
| HCC16 | NBNC | Male | 101 | 146 | 11 | 36 | 294 |
| HCC72 | HBV | Male | 66 | 185 | 12 | 27 | 290 |
| HCC17 | HCV | Male | 78 | 178 | 3 | 23 | 282 |
| HCC66 | NBNC | Male | 40 | 186 | 31 | 18 | 275 |
| HCC11 | HCV | Male | 64 | 165 | 4 | 31 | 264 |
| HCC63 | HCV | Male | 52 | 166 | 33 | 13 | 264 |
| HCC14 | NBNC | Male | 26 | 205 | 7 | 22 | 260 |
| HCC04 | HBV | Male | 41 | 195 | 2 | 20 | 258 |
| HCC55 | HBV | Male | 58 | 170 | 17 | 13 | 258 |
| HCC64 | NBNC | Male | 46 | 186 | 11 | 12 | 255 |
| HCC13 | HCV | Male | 57 | 152 | 4 | 15 | 228 |
| HCC61 | HBV | Male | 45 | 154 | 10 | 15 | 224 |
| HCC58 | HCV | Female | 22 | 161 | 14 | 11 | 208 |
| HCC45 | HBV | Female | 15 | 127 | 36 | 16 | 194 |
| HCC12 | NBNC | Female | 86 | 79 | 11 | 13 | 189 |
| HCC19 | NBNC | Female | 18 | 141 | 3 | 13 | 175 |
| HCC09 | HCV | Male | 22 | 127 | 2 | 21 | 172 |
| HCC23 | HBV | Female | 16 | 103 | 29 | 21 | 169 |
| HCC18 | NBNC | Female | 22 | 122 | 3 | 12 | 159 |
| HCC07 | HCV | Male | 38 | 105 | 1 | 14 | 158 |
| HCC10 | HCV | Male | 25 | 113 | 1 | 18 | 157 |
| HCC60 | HCV | Female | 24 | 105 | 4 | 11 | 144 |
| HCC71 | HBV | Male | 26 | 100 | 7 | 8 | 141 |
| HCC03 | HCV | Female | 21 | 103 | 1 | 5 | 130 |
| HCC21 | HCV | Male | 10 | 102 | 0 | 18 | 130 |
| HCC32 | HBV | Male | 11 | 91 | 3 | 15 | 120 |
| HCC20 | NBNC | Male | 9 | 96 | 2 | 11 | 118 |
| HCC53 | HCV | Male | 17 | 74 | 6 | 8 | 105 |
| HCC35 | NBNC | Male | 6 | 86 | 5 | 5 | 102 |
| HCC54 | NBNC | Male | 4 | 88 | 0 | 10 | 102 |
| HCC24 | HBV | Male | 0 | 79 | 5 | 15 | 99 |
| HCC02 | HCV | Male | 11 | 81 | 1 | 5 | 98 |
| HCC65 | HBV | Male | 3 | 71 | 4 | 11 | 89 |
| HCC15 | NBNC | Male | 8 | 67 | 0 | 6 | 81 |
| HCC33 | HBV | Male | 14 | 56 | 2 | 7 | 79 |
| HCC56 | NBNC | Male | 5 | 57 | 4 | 2 | 68 |
| HCC67 | HBV | Male | 2 | 46 | 1 | 4 | 53 |
| HCC50 | HBV | Female | 3 | 37 | 1 | 3 | 44 |
| All | | | 102113 | 11017 | 928 | 1134 | 115192 |

**Table S4.** Chromosomal landscape of structural variation number

All HCC samples

| Chr | DEL | INS | INV | DUP | Total |
| --- | --- | --- | --- | --- | --- |
| 1 | 84177 | 4687 | 701 | 504 | 90069 |
| 2 | 6842 | 1450 | 67 | 128 | 8487 |
| 3 | 3559 | 482 | 45 | 27 | 4113 |
| 4 | 474 | 309 | 15 | 28 | 826 |
| 5 | 702 | 370 | 7 | 52 | 1131 |
| 6 | 1442 | 405 | 24 | 37 | 1908 |
| 7 | 247 | 344 | 4 | 41 | 636 |
| 8 | 376 | 357 | 3 | 50 | 786 |
| 9 | 710 | 181 | 13 | 11 | 915 |
| 10 | 341 | 424 | 10 | 50 | 825 |
| 11 | 278 | 313 | 9 | 53 | 653 |
| 12 | 436 | 215 | 2 | 24 | 677 |
| 13 | 112 | 43 | 6 | 1 | 162 |
| 14 | 138 | 22 | 1 | 0 | 161 |
| 15 | 9 | 69 | 1 | 0 | 79 |
| 16 | 76 | 182 | 4 | 27 | 289 |
| 17 | 208 | 345 | 1 | 22 | 576 |
| 18 | 564 | 114 | 4 | 2 | 684 |
| 19 | 390 | 315 | 1 | 26 | 732 |
| 20 | 628 | 128 | 8 | 19 | 783 |
| 21 | 12 | 32 | 0 | 1 | 45 |
| 22 | 278 | 48 | 1 | 1 | 328 |
| X | 70 | 152 | 1 | 30 | 253 |
| Y | 44 | 30 | 0 | 0 | 74 |
| All | 102113 | 11017 | 928 | 1134 | 115192 |

HBV-related HCC

| Chr | DEL | INS | INV | DUP | Total |
| --- | --- | --- | --- | --- | --- |
| 1 | 81446 | 2958 | 516 | 304 | 85224 |
| 2 | 4959 | 907 | 46 | 82 | 5994 |
| 3 | 3171 | 274 | 22 | 13 | 3480 |
| 4 | 367 | 181 | 10 | 14 | 572 |
| 5 | 686 | 244 | 6 | 32 | 968 |
| 6 | 1335 | 209 | 18 | 19 | 1581 |
| 7 | 184 | 169 | 1 | 17 | 371 |
| 8 | 369 | 246 | 2 | 38 | 655 |
| 9 | 574 | 101 | 10 | 8 | 693 |
| 10 | 303 | 244 | 9 | 23 | 579 |
| 11 | 253 | 176 | 7 | 24 | 460 |
| 12 | 391 | 125 | 1 | 13 | 530 |
| 13 | 81 | 23 | 5 | 1 | 110 |
| 14 | 127 | 14 | 0 | 0 | 141 |
| 15 | 6 | 49 | 0 | 0 | 55 |
| 16 | 65 | 110 | 4 | 13 | 192 |
| 17 | 202 | 183 | 1 | 15 | 401 |
| 18 | 539 | 83 | 2 | 2 | 626 |
| 19 | 374 | 156 | 1 | 11 | 542 |
| 20 | 537 | 91 | 7 | 16 | 651 |
| 21 | 7 | 13 | 0 | 0 | 20 |
| 22 | 221 | 36 | 1 | 1 | 259 |
| X | 61 | 99 | 1 | 21 | 182 |
| Y | 42 | 16 | 0 | 0 | 58 |
| All | 96300 | 6707 | 670 | 667 | 104344 |

HCV-related HCC

| Chr | DEL | INS | INV | DUP | Total |
| --- | --- | --- | --- | --- | --- |
| 1 | 369 | 635 | 60 | 83 | 1147 |
| 2 | 43 | 199 | 3 | 20 | 265 |
| 3 | 33 | 99 | 9 | 5 | 146 |
| 4 | 6 | 56 | 2 | 6 | 70 |
| 5 | 4 | 35 | 0 | 7 | 46 |
| 6 | 16 | 93 | 0 | 9 | 118 |
| 7 | 15 | 94 | 1 | 18 | 128 |
| 8 | 2 | 73 | 0 | 7 | 82 |
| 9 | 12 | 28 | 0 | 2 | 42 |
| 10 | 15 | 90 | 0 | 17 | 122 |
| 11 | 14 | 76 | 1 | 22 | 113 |
| 12 | 2 | 47 | 0 | 4 | 53 |
| 13 | 2 | 6 | 0 | 0 | 8 |
| 14 | 3 | 5 | 0 | 0 | 8 |
| 15 | 2 | 10 | 1 | 0 | 13 |
| 16 | 6 | 43 | 0 | 5 | 54 |
| 17 | 5 | 101 | 0 | 6 | 112 |
| 18 | 0 | 11 | 0 | 0 | 11 |
| 19 | 0 | 43 | 0 | 5 | 48 |
| 20 | 1 | 18 | 1 | 3 | 23 |
| 21 | 1 | 7 | 0 | 0 | 8 |
| 22 | 0 | 7 | 0 | 0 | 7 |
| X | 2 | 11 | 0 | 3 | 16 |
| Y | 1 | 6 | 0 | 0 | 7 |
| All | 554 | 1793 | 78 | 222 | 2647 |

NBNC-related HCC

| Chr | DEL | INS | INV | DUP | Total |
| --- | --- | --- | --- | --- | --- |
| 1 | 2362 | 1094 | 125 | 117 | 3698 |
| 2 | 1840 | 344 | 18 | 26 | 2228 |
| 3 | 355 | 109 | 14 | 9 | 487 |
| 4 | 101 | 72 | 3 | 8 | 184 |
| 5 | 12 | 91 | 1 | 13 | 117 |
| 6 | 91 | 103 | 6 | 9 | 209 |
| 7 | 48 | 81 | 2 | 6 | 137 |
| 8 | 5 | 38 | 1 | 5 | 49 |
| 9 | 124 | 52 | 3 | 1 | 180 |
| 10 | 23 | 90 | 1 | 10 | 124 |
| 11 | 11 | 61 | 1 | 7 | 80 |
| 12 | 43 | 43 | 1 | 7 | 94 |
| 13 | 29 | 14 | 1 | 0 | 44 |
| 14 | 8 | 3 | 1 | 0 | 12 |
| 15 | 1 | 10 | 0 | 0 | 11 |
| 16 | 5 | 29 | 0 | 9 | 43 |
| 17 | 1 | 61 | 0 | 1 | 63 |
| 18 | 25 | 20 | 2 | 0 | 47 |
| 19 | 16 | 116 | 0 | 10 | 142 |
| 20 | 90 | 19 | 0 | 0 | 109 |
| 21 | 4 | 12 | 0 | 1 | 17 |
| 22 | 57 | 5 | 0 | 0 | 62 |
| X | 7 | 42 | 0 | 6 | 55 |
| Y | 1 | 8 | 0 | 0 | 9 |
| All | 5259 | 2517 | 180 | 245 | 8201 |

**Table S5**. Distribution and proportion of repetitive elements across structural variation types.

| Hepatitis | Type | DEL | | INS | | INV | | DUP | | Total | |
| --- | --- | --- | --- | --- | --- | --- | --- | --- | --- | --- | --- |
|  |  | Number | Ratio | Number | Ratio | Number | Ratio | Number | Ratio | Number | Ratio |
|  | SINEs | 24155 | 35.72% | 479 | 18.78% | 457 | 27.12% | 1142 | 37.60% | 26233 | 35.03% |
|  | LINEs | 22259 | 32.92% | 684 | 26.81% | 542 | 32.17% | 882 | 29.04% | 24367 | 32.54% |
|  | LTR elements | 8975 | 13.27% | 276 | 10.82% | 223 | 13.23% | 335 | 11.03% | 9809 | 13.10% |
|  | Simple_repeat | 6324 | 9.35% | 817 | 32.03% | 247 | 14.66% | 409 | 13.47% | 7797 | 10.41% |
| HBV | DNA elements | 4526 | 6.69% | 79 | 3.10% | 162 | 9.61% | 208 | 6.85% | 4975 | 6.64% |
|  | Low_complexity | 1052 | 1.56% | 92 | 3.61% | 45 | 2.67% | 35 | 1.15% | 1224 | 1.63% |
|  | Retroposon/SVA | 203 | 0.30% | 79 | 3.10% | 0 | 0.00% | 6 | 0.20% | 288 | 0.38% |
|  | Small RNA | 91 | 0.13% | 3 | 0.12% | 9 | 0.53% | 9 | 0.30% | 112 | 0.15% |
|  | Satellites | 30 | 0.04% | 42 | 1.65% | 0 | 0.00% | 11 | 0.36% | 83 | 0.11% |
|  | ALL | 67615 | 100.00% | 2551 | 100.00% | 1685 | 100.00% | 3037 | 100.00% | 74888 | 100.00% |
|  | SINEs | 638 | 31.15% | 99 | 14.75% | 45 | 27.61% | 249 | 33.74% | 1031 | 28.48% |
|  | LINEs | 548 | 26.76% | 163 | 24.29% | 55 | 33.74% | 234 | 31.71% | 1000 | 27.62% |
|  | LTR elements | 304 | 14.84% | 80 | 11.92% | 25 | 15.34% | 70 | 9.49% | 479 | 13.23% |
|  | Simple_repeat | 257 | 12.55% | 239 | 35.62% | 19 | 11.66% | 132 | 17.89% | 647 | 17.87% |
| HCV | DNA elements | 257 | 12.55% | 22 | 3.28% | 14 | 8.59% | 38 | 5.15% | 331 | 9.14% |
|  | Low_complexity | 32 | 1.56% | 22 | 3.28% | 5 | 3.07% | 10 | 1.36% | 69 | 1.91% |
|  | Retroposon/SVA | 4 | 0.20% | 31 | 4.62% | 0 | 0.00% | 0 | 0.00% | 35 | 0.97% |
|  | Small RNA | 7 | 0.34% | 2 | 0.30% | 0 | 0.00% | 5 | 0.68% | 14 | 0.39% |
|  | Satellites | 1 | 0.05% | 13 | 1.94% | 0 | 0.00% | 0 | 0.00% | 14 | 0.39% |
|  | ALL | 2048 | 100.00% | 671 | 100.00% | 163 | 100.00% | 738 | 100.00% | 3620 | 100.00% |
|  | SINEs | 1224 | 30.30% | 173 | 16.98% | 212 | 28.46% | 377 | 38.04% | 1986 | 29.23% |
|  | LINEs | 1400 | 34.65% | 259 | 25.42% | 234 | 31.41% | 256 | 25.83% | 2149 | 31.63% |
|  | LTR elements | 580 | 14.36% | 108 | 10.60% | 111 | 14.90% | 93 | 9.38% | 892 | 13.13% |
|  | Simple_repeat | 442 | 10.94% | 364 | 35.72% | 84 | 11.28% | 168 | 16.95% | 1058 | 15.57% |
| NBNC | DNA elements | 312 | 7.72% | 36 | 3.53% | 88 | 11.81% | 62 | 6.26% | 498 | 7.33% |
|  | Low_complexity | 59 | 1.46% | 32 | 3.14% | 16 | 2.15% | 14 | 1.41% | 121 | 1.78% |
|  | Retroposon/SVA | 11 | 0.27% | 31 | 3.04% | 0 | 0.00% | 1 | 0.10% | 43 | 0.63% |
|  | Small RNA | 10 | 0.25% | 5 | 0.49% | 0 | 0.00% | 5 | 0.50% | 20 | 0.29% |
|  | Satellites | 2 | 0.05% | 11 | 1.08% | 0 | 0.00% | 15 | 1.51% | 28 | 0.41% |
|  | ALL | 4040 | 100.00% | 1019 | 100.00% | 745 | 100.00% | 991 | 100.00% | 6795 | 100.00% |

**Table S6.** Sequences of primers used in this study.

| Primer name | species | Sense (5'-3') |
| --- | --- | --- |
| HCC29-DPRX-F | human | CCTGGCATTGGGGTATGGTA |
| HCC29-DPRX-R | human | AACAAGGATCACTGGCCAGA |
| HCC46-DPRX-F | human | AGAGTCTCGCTTTGTCACCA |
| HCC46-DPRX-R | human | CTGAACCTTTACCCCGTTGC |
| HCC51-DPRX-F | human | TTCAGCAATGTCAACGACCG |
| HCC51-DPRX-R | human | TCAATAGTTCGCCCAGGGAG |
| HCC59-PIM3-1-F | human | TCTGGACCTTTACCCCGTTG |
| HCC59-PIM3-1-R | human | ACTCCCCTGTTCAGCTCTTC |
| HCC59-PIM3-2-F | human | ATACTGCGGAACTCCTAGCC |
| HCC59-PIM3-2-R | human | TTTCATCATGTTGGCCAGGC |
